# miR-20487-5p/SERCA1/MAPK/ERK Pathway Regulates Newt Limb Regeneration

**DOI:** 10.3390/biology15141107

**Published:** 2026-07-09

**Authors:** Lin Zhu, Dan Zhang, Zongping Li, Hongxiao Sun, Mengdi Cheng, Jie Tang, Liyuan Jia, Xuli Liu, Fulin Chen, Hong Tan

**Affiliations:** 1 Laboratory of Tissue Engineering, Provincial Key Laboratory of Biotechnology, Key Laboratory of Resource Biology and Biotechnology in Western China, Ministry of Education, College of Life Sciences, Northwest University, Xi’an 710069, China; 2 Shaanxi Institute of Zoology, Xi’an 710032, China; 3 Department of Gastroenterology, Zhuzhou Hospital Affiliated to Central South University, Zhuzhou 412007, China

**Keywords:** SERCA1, myotube dedifferentiation, blastema formation, limb regeneration

## Abstract

*Cynops orientalis*, a member of the Salamandridae family, can regenerate severely injured limbs throughout its lifespan. The molecular mechanisms regulating limb regeneration in *C. orientalis* remain largely unknown. SERCA1 (sarcoplasmic/endoplasmic reticulum calcium ATPase 1) is a major sarco/endoplasmic reticulum calcium pump in skeletal muscle, and our previous transcriptomic sequencing results showed increased SERCA1 mRNA expression during the early stages of newt limb regeneration. In the present study, we investigated the role and regulatory mechanism of SERCA1 during newt limb regeneration by using a limb-amputation model, shRNA-mediated knockdown, Thapsigargin-mediated SERCA1 inhibition, miRNAomics analysis, dual-luciferase reporter assays and Agomir therapy. Knockdown of SERCA1 expression or suppression of SERCA1 activity impaired skeletal muscle cell dedifferentiation, disrupted AEC and blastema formation, reduced cell proliferation, and significantly delayed limb regeneration compared with control animals. Through miRNAomics analysis and in vitro dual-luciferase reporter assays, we identified miR-20487-5p as a negative regulator of SERCA1 expression in *C. orientalis*. In vivo treatment with miR-20487-5p Agomir down-regulated SERCA1 expression resulted in limb-regenerative defects. Mechanistically, shRNA-SERCA1 or miR-20487-5p Agomir treatment reduced ERK phosphorylation in regenerating limb tissue, suggesting an association between the miR-20487-5p/SERCA1 axis and MAPK/ERK activation. Taken together, our study supports the significant role of the miR-20487-5p/SERCA1/MAPK/ERK axis in early newt limb regeneration. This study expands the current understanding of molecular mechanisms initiating newt limb regeneration.

## Simple Summary

Newts possess powerful regenerative capacity and can regrow amputated limbs. However, the molecular mechanisms modulating this process remain largely unknown. Here, we found that SERCA1 was highly expressed during early limb regeneration. Suppression of SERCA1 expression or enzyme activity impaired AEC and blastema formation, reduced proliferating cells, and delayed limb regeneration. A novel miRNA, miR-20487-5p, was identified and experimentally supported as a negative regulator of SERCA1 expression. Functional experiments showed that miR-20487-5p Agomir treatment suppressed SERCA1 expression and produced similar regenerative defects. Further assays suggested that MAPK/ERK activation was associated with this regulatory axis. This work provides evidence for a miR-20487-5p/SERCA1/MAPK/ERK regulatory axis that participates in the newt limb-regeneration process.

## 1. Introduction

Urodele amphibians possess powerful regeneration capacity by which amputated appendages can be fully reconstituted anatomically and functionally. Upon injury, the wound epidermis (WE) is rapidly formed by migrating epithelial cells within 24 h, and subsequently thickens into the apical epidermal cap (AEC) [1,2]. Starting from the first week till the 3rd or 4th week of regeneration, the interactions among AEC, nerve and residual cells initiate blastema formation, a process in which local mature cells, such as skeletal muscle cells, dermal cells, Schwann cells and chondrocytes, dedifferentiate and gather to form a collection of heterogenetic lineage-restricted progenitor cells [3,4]. The blastema structure proceeds to differentiate and proliferate and eventually re-develops into a replica of the missing part of the limb within 5–7 weeks [5,6]. It is of special interest for researchers to unravel the exquisite cellular and molecular machinery triggering the regeneration initiation event. However, the key molecules and mechanisms remain largely unclear.

Skeletal muscle cells are one of the major cell types contributing to blastema formation, which go through dedifferentiation into progenitor cells during the early newt limb-regeneration process [7]. Cell lineage tracing of newt limb regeneration showed that multinucleated myofibres fragmented into mononucleate progenitor cells, down-regulated muscle-specific proteins, and then re-entered the cell cycle to dedifferentiate into multipotent blastema cells [8,9,10]. Studies have revealed key roles of multiple transcription factors and signaling proteins in regulating the plasticity of skeletal muscle cells. For example, in axolotl limb regeneration, the pluripotent muscle satellite cells that were activated to re-enter the cell cycle during blastema formation were found to express *Pax7* [11]. A MARCKS-like protein, a protein kinase C substrate protein, was demonstrated to play a critical role in inducing axolotl myotube cell cycle re-entry [12,13,14]. Interestingly, Wang et al. elaborated that fragmentation of newt myotubes was initiated by programmed cell death, which was then interrupted and diverted into the dedifferentiation path [15]. A recent study showed that exogenous transplantation of senescent newt A1 cells promoted myotube cell cycle re-entry and dedifferentiation in newt limb regeneration by activating the FGF-ERK pathway [14].

In our previous work of transcriptomic analysis during limb regeneration of *C. orientalis* [16], we noticed a rapid increase in SERCA1 expression upon limb amputation, and the increase persisted throughout the blastema formation period. Therefore, we hypothesized that SERCA1 participated in the early events of newt limb regeneration. SERCA1, encoded by *ATP2a1*, is a highly conserved calcium ion channel enzyme residing on the sarco/endoplasmic reticulum (SR) membrane and is mainly expressed in fast-twitch skeletal muscle [17,18]. It is a major regulator of intracellular Ca^2+^ homeostasis that actively transfers Ca^2+^ ions from the cytosol into the lumen of SR and is responsible for skeletal muscle relaxation. The SR is a delicate membranous structure that functions as the major intracellular Ca^2+^ storage organelle. The SERCA1 protein is a cap-like structure formed by the actuator (A), phosphorylation (P), nucleotide-binding (N) domains and a transmembrane (M) domain [19]. The P domain hydrolyzes nucleotides to release free Pi; the A domain connects to the transmembrane domain and triggers the Ca^2+^ channel to allow cytoplasmic Ca^2+^ to enter the calcium-binding sites; and the N domain releases ADP that drives the calcium ions’ uptake by the SR lumen [20,21]. Intracellular Ca^2+^ flux conveys bioelectric signaling capable of regulating a variety of cell behaviors, such as cell migration, proliferation, differentiation, transcription activities, and so on [22,23,24]. Recent studies suggest bioelectrical gradients and ion flow patterns play vital roles during regeneration processes in multiple organisms [25,26,27,28,29]. Nevertheless, little is known about the intracellular Ca^2+^ homeostasis, controlled by the Ca^2+^ channel pump, which affects appendage regeneration, in particular, initiation of the regeneration process.

To investigate the role of SERCA1 in the newt limb-regeneration process, we utilized the newt limb-amputation model in which knockdown of SERCA1 expression or inhibition of SERCA1 activity disrupted blastema formation and sabotaged newt limb-regeneration. Further investigation demonstrated that the pattern of skeletal muscle cell fragmentation, blastema formation and ECM remodeling in the early regeneration stage were disrupted in SERCA1-suppressed animal groups. By conducting miRNA–mRNA transcriptome pairing analysis and in vitro luciferase assay, we identified and confirmed a novel microRNA, miR-20487-5p_611 (hereafter referred to as miR-20487-5p), which targeted and regulated SERCA1 post-transcriptionally. Functionally, administration with miR-20487-5p Agomir down-regulated SERCA1 expression showed significantly delayed newt limb-regeneration. Bioinformatic KEGG analysis of DE (differential expressed) miRNAs targeted mRNAs, indicating enrichment of the MAPK/ERK (mitogen-activated protein kinase) pathway during early stages of the newt limb-regeneration process. Through shRNA-SERCA1 or miR-20487-5p Agomir treatment in the newt limb-regeneration model, our results revealed markedly suppressed ERK phosphorylation levels compared with control animals, indicating that MAPK/ERK activation was associated with the miR-20487-5p/SERCA1 pathway.

Together, this study was designed to investigate the significant role of the miR-20487-5p/SERCA1/MAPK/ERK axis in early newt limb regeneration. Our study expands the current understanding of the newt limb-regeneration initiation mechanism and paves the way towards knowledge for regenerative medicine development.

## 2. Materials and Methods

### 2.1. Animals and Procedures

All experiments were performed according to the ethical policies and procedures approved by the Northwest University Animal Care and Use Committee (Approval No. NWU-AWC-20240903S). Adult Chinese fire-bellied newts (*Cynops orientalis*), 6–8 cm in body length, were purchased from a commercial farm in Hubei, China. Animals were acclimated before experiments and maintained in clean water at 20 °C under standard laboratory conditions, with regular water changes and bloodworm feedings.

Surgical procedures were performed on newts to create a limb-regeneration model. Before operation, animals were soaked in gentamicin sulfate salt solution (Solarbio, Beijing, China) for 2 h, and anesthetized by 0.1% Tricaine methane sulfonate (Sigma–Aldrich, St. Louis, MO, USA). Then, the right forelimb amputations at the midstylopod level on newts were conducted using surgery scissors under a dissecting microscope (Model SZX10, OLYMPUS, Tokyo, Japan). Animals were placed back into clean water after the procedure. Proximal regenerating tissues were collected at 0 h and 1, 3, 7, 14, 30 and 42 days post amputation (dpa) for subsequent assays as illustrated in Supplementary Scheme S1A. These time points represent key stages during newt limb regeneration: wound healing (1 dpa), AEC formation (3 dpa), blastema formation (7 dpa), blastema cell proliferation (14 dpa), pattern formation (30 dpa) and digits formation (42 dpa). At each time point, 15 newts were randomly divided into 3 groups and sample tissues within each group were pooled for qRT-PCR and WB analysis.

For electroporation of shRNA-SERCA1 into limb tissue, the plasmid was generated by cloning shRNA-SERCA1 into a pDsRed-shRNA vector (Supplementary Figure S1A), which was modified by cloning a U6 promoter in the pDsRed-Express2-C1 vector (Cat# 632538, Clontech, Mountain View, CA, USA) to drive shRNA expression in newt cells. Newts were randomly divided into 2 groups (the shRNA-SERCA1 treated group and the vector treated group). Firstly, animals were anesthetized with 0.1% tricaine (SigmaAldrich, St. Louis, MO, USA), and then 10 μL (500 ng/μL) of the plasmid was injected intramuscularly at multiple sites surrounding the midstylopod level of the newt forelimb using a microinjector. Then, the shRNA-SERCA1 plasmid was electroporated with 5 pulses (voltage 25 V, pulse width 100 ms, interval 200 ms) using a Scientz-2C electroporation machine (Ningbo Scientz, Ningbo, China), as illustrated in Supplementary Scheme S1B. Animals were allowed to recover in sterile water and limb amputation was performed 3 days post electroporation.

For the SERCA1 inhibitor assay, animals were randomly divided into 2 groups (the Thapsigargin (TargetMol, Boston, MA, USA)—treated group and the DMSO (SigmaAldrich, St. Louis, MO, USA)—treated group). The Thapsigargin group were soaked in 0.1 μM Thapsigargin dissolved in 0.01% DMSO solution. Animals were kept in the presence of Thapsigargin/DMSO or 0.01% DMSO with fresh solution changed every two days until the samples were collected. The limb amputation was conducted on the 3rd day of inhibitor treatment and proximal regenerating tissues were collected at 0 h and 3, 7, 14, 30 and 42 days post amputation (dpa) as illustrated in Supplementary Scheme S1A. 

For in vivo miR-20487-5p Agomir (10 μL; sequence: 5′-CGCCAGGGGCUGUAGGCAUU-3′; RiboBio, Guangzhou, China) treatment, newts were randomly divided into the miR-20487-5p Agomir-treated group and the negative control (NC) group. A chemically synthesized mature miR-20487-5p Agomir at a concentration of 0.05 nM was injected intramuscularly around the amputation site 2 h after forelimb amputation at the midstylopod level and was administered every 4 days until 12 days post-amputation. NC Agomir (sequence: 5′-TAACACGTCTATACCCCA-3′; RiboBio, Guangzhou, China) was used as the control.

### 2.2. Tissue Sectioning and Histology

Limb samples were collected and fixed in 4% paraformaldehyde (PFA, Yongda, Tianjin, China) at 4 °C for 12 h, decalcified with 12% EDTA (Solarbio, Beijing, China) for 2 weeks and embedded in paraffin (Hualing, Shanghai, China). Tissue was sectioned at 5 μm. After dehydration with gradient ethanol, the sections were stained with Hematoxylin-eosin (Solarbio, Beijing, China). Masson’s trichrome (Solarbio, Beijing, China) staining was performed to assess collagen deposition in regenerated tissue, and Senna-Solid Green (Solarbio, Beijing, China) staining was conducted to observe cartilage regeneration.

### 2.3. Immunofluorescence Staining

For analysis of skeletal muscle dedifferentiation and SERCA1 deposition, double immunofluorescence staining of SERCA1 with myosin heavy chain (MF20, Developmental Studies Hybridoma Bank, Iowa City, IA, USA) was performed. The paraffin sections were dewaxed and dehydrated with a series of gradient alcohol washes. After antigen retrieval (sodium citrate (Tianli, Tianjin, China) 2.4 g/L, citric acid (Tianli, Tianjin, China) 0.4g/L, pH = 6), slides were blocked with 5% BSA (Solarbio, Beijing, China) for 1 h. Rabbit anti-SERCA1 (1:100, Immunoway, San Jose, CA, USA) and mouse anti-MF20 (1:100) were incubated overnight at 4 °C. Goat anti-rabbit IgG (Alexa Fluor 488,1:300, Abcam, Cambridge, UK) and goat anti-mouse IgG (Alexa Fluor 594, 1:300, Abcam, Cambridge, UK) secondary antibodies were incubated for 1 to 4 h at room temperature. Nuclei were stained with DAPI (Beyotime, Haimen, China) for 8 min. For quantification of SERCA1-positive cells and MF20-positive cells, the percentage of positive cells out of total DAPI cells was measured using ImageJ (version 1.54f).

### 2.4. BrdU Analysis

BrdU analysis was conducted to measure proliferating cells. 8 mg/mL (20 μL per animal) were administered by intraperitoneal injection before limb amputation. Limb tissues were collected at regeneration time points, and then embedded in paraffin wax for sectioning into 5-micron sections. The sections were immersed in a solution of 1 mol/L HCl (Tianli, Tianjin, China) at 37 °C for 15 min and fixed in 4% PFA (Yongda, Tianjin, China) for 10 min. The primary antibody was BrdU Mouse mAb (ABclonal, Wuhan, China). The secondary antibody used goat anti-mouse IgG (Alexa Fluor488) (1:1000, Abcam, Cambridge, UK). Confocal microscopy (Model LV3000, Olympus Corporation, Tokyo, Japan) was performed to obtain images. For total BrdU quantification, the percentage of BrdU cells out of total DAPI cells across regenerative tissue was calculated using ImageJ (*n* = 3).

### 2.5. qRT-PCR Analysis

Regenerating limb tissue was collected and grinded using the homogenizer (Jingxin Technology, Shanghai, China). Tissues harvested from five animals were mixed as one biological sample, and three biological replicates were used per group. Total RNA was isolated using TRIzol Reagent (Invitrogen, Carlsbad, CA, USA) with a tissue grinder (70 Hz, 12 min, Jingxin Technology, Shanghai, China). The concentration and purity of RNA were measured with a Denovix NanoDrop spectrophotometer (Denovix, Wilmington, DE, USA). Total RNA was reverse-transcribed into cDNA using the Transcriptor First Strand cDNA Synthesis Kit (Roche, Basel, Switzerland) and the reverse transcription program was 37 °C for 60 min and 85 °C for 5 min. qRT-PCR was performed with SYBR Premix Ex Taq (TaKaRa Bio, Kusatsu, Shiga, Japan) on a Bio-Rad real-time fluorescence quantitative PCR system (Bio-Rad, Hercules, CA, USA), with pre-denaturation at 95 °C for 30 s, followed by 40 cycles of 95 °C for 5 s, 60 °C for 15 s and 72 °C for 10 s, with GAPDH serving as the endogenous reference gene.

For miRNA qRT-PCR analysis, cDNA was reverse-transcribed using the miRNA First Strand cDNA Synthesis (Tailing Reaction) Kit (Cat. # B532451-0050, Sangon Biotech, Shanghai, China). Quantitative real-time PCR was performed using the 2× qPCR Premix (SYBR Green) (Cat. # CM0139, Aikerui Biotech, Changsha, China) on the real-time fluorescence quantitative PCR detection system (Bio-Rad, Hercules, CA, USA) at 95 °C for 10 min, followed by 40 cycles of 95 °C for 10 s, 60 °C for 20 s and 72 °C for 10 s. U6 snRNA was selected as the internal reference for normalization of miRNA expression. A total of 3 technical replicates were performed per sample. The relative expression of each gene was calculated with the 2^−ΔΔCt^ method. Primer sets were listed in Supplementary Table S1.

### 2.6. Enzyme Activity Assay

The enzyme activity of SERCA1 was measured by quantification of the amount of free phosphate released during the enzyme reaction, utilizing a malachite green phosphate assay kit (Sigma, St. Louis, MO, USA). The reaction was initiated by the addition of ATP and conducted at 37 °C. Samples were collected at 0, 15, and 30 min, with 60 μL of the reaction mixture being removed and added to 240 μL of pre-cooled dilution solution at each time point. The reaction was terminated at −80 °C, and absorbance was measured at 620 nm. A standard phosphate curve ($y=0.01436x+0.175,R^{2}=0.9965$) was prepared in enzyme buffer according to the manufacturer’s instructions to determine the amount of free phosphate released by SERCA1.

### 2.7. Bioinformatic Analysis of miRNA-SERCA1 mRNA Pairs

To predict the miRNAs regulating SERCA1 expression, the miRNA and mRNA transcriptomic-sequencing raw data from our previously published work was analyzed [16]. Briefly, the total mRNA and small RNA libraries of newt limb regeneration at continuous time points were prepared using the mRNA-Seq sample preparation kit (Illumina, San Diego, CA, USA) and Small RNA Sample Prep Kits (Illumina, San Diego, CA, USA), respectively, followed by sequencing using an Illumina Hiseq 2500 conducted by LC-BIO (Hangzhou, China). The resulting transcriptome was assembled de novo, and miRNA annotation was based on miRbase 21.0 database.

For prediction of potential miRNAs targeting SERCA1 mRNA, TargetScan (v3.3a) and miRanda (Release 8.0) were used to analyze DE miRNAs (fold change ≥ 2 or ≤0.5, and *p*-value < 0.05) and SERCA1 mRNA data (0, 3, 7, 14, 28 and 42 dpa). miRNAs with predicted binding sites in the CDS region of SERCA1 mRNA were screened. An alluvial plot was constructed to visualize negatively correlated miRNA-SERCA1 mRNA pairs using R (v4.3.0) and the ggalluvial package (v0.12.3). For enhanced clarity of the co-regulatory architecture, flow widths were normalized to a uniform weighting strategy. KEGG pathways enriched with DE miRNA target genes were annotated using the R clusterProfiler package (R v4.x, Bioconductor, Guangzhou, China; https://bioconductor.org/packages/clusterProfiler/), with significance defined by a Benjamini–Hochberg adjusted *p* < 0.05.

### 2.8. Dual-Luciferase Reporter Assay (DLR)

Newt SERCA1 mRNA containing miR-20487-5p-binding sequences were amplified from cDNA and cloned into psiCHECK-2 vector (Promega, Madison, WI, USA). Binding-region mutations were created with a Q5 Site-Directed Mutagenesis Kit (New England Biolabs, Ipswich, MA, USA). Primers used to construct plasmids are shown in Supplementary Table S1.

Luciferase assays were then performed using the DLR assay system (Promega, Madison, WI, USA) as described previously [16]. Briefly, transient transfection of 293T cells (4 × 10^4^ cells per well) was carried out in 24-well plates with Lipofectamine 2000 (Invitrogen, Carlsbad, CA, USA) under manufacturer’s instructions. The cells were co-transfected with 500 ng of luciferase reporter constructs containing the wild-type or mutant SERCA1 3′-UTR plasmid and 15 pmol of the miR-20487-5p mimics or control mimics (GeneCopoeia, Guangzhou, China) by Lipofectamine 2000 (Invitrogen, Carlsbad, CA, USA). Cells were lysed 24 h following transfection, and then the Renilla luciferase (RLuc) and firefly luciferase (Fluc) activities were assessed by using a Synergy 2 luminometer (BioTek, Winooski, VT, USA). Rluc signals were normalized to the intraplasmid Fluc transfection control (*n* = 3).

### 2.9. Western Blotting Analysis

Limb tissue was ground using a homogenizer and lysed in RIPA buffer (Beyotime, Haimen, China) on ice. The homogenate was centrifuged at 12,000 rpm for 5 min at 4 °C, and the supernatant was harvested for total protein extraction. Protein concentration was measured using a BCA protein assay kit (Thermo Scientific, Waltham, MA, USA). All samples were diluted to 2.0 μg/μL. Protein samples were separated by SDS-PAGE (80 V for 30 min, then 120 V for 1 h) and then electrotransferred to PVDF membranes (Millipore, Burlington, MA, USA) at 230 mA for 2 h in an ice bath. Membranes were blocked with 5% skim milk in PBST for 1 h at room temperature. The following primary antibodies were used: rabbit anti-SERCA1 (Cat# YT4253, 1:1000, Immunoway, San Jose, CA, USA), rabbit anti-ERK (1:1000, Abmart, Shanghai, China), rabbit anti-p-ERK (1:1000, Abmart, Shanghai, China), and rabbit anti-β-actin (1:1000, Abmart, Shanghai, China). Primary antibodies were incubated at 4 °C overnight. After being rinsed in PBST three times for 5 min, membranes were incubated with goat anti-rabbit IgG (H + L) secondary antibody (1:5000, Immunoway, San Jose, CA, USA). Signals were detected using BeyoECL Plus chemiluminescence reagent (Beyotime, Haimen, China), and band intensities were analyzed using ImageJ.

### 2.10. Imaging Acquisition

The Biological Microscope (Model BX53, Evident Corporation, Nagano, Japan) and the Digital Slice Scanning System were used to acquire HE, Masson’s Trichrome and Senna-Solid Green staining images. Imaging of immunofluorescence staining and BrdU assays was performed by using the Olympus confocal microscope (Model LV3000, Olympus Corporation, Tokyo, Japan).

### 2.11. Statistical Analyses

Statistical analyses were performed using SPSS 20.0 and GraphPad Prism 8. For qRT-PCR assays, each biological sample was analyzed with technical replicates. For histological and immunofluorescence analyses, *n* indicates independent biological samples. Data are presented as mean ± SD. Independent samples’ *t*-test or one-way ANOVA was performed as indicated, and *p* < 0.05 was considered statistically significant.

### 2.12. Data Availability

The microRNA sequencing raw data have been deposited in the NCBI Sequence Read Archive (SRA, https://www.ncbi.nlm.nih.gov/sra, accessed on 28 June 2026) under accession number PRJNA1479149.

## 3. Results

### 3.1. SERCA1 Expression and Enzymatic Activity Increased During Early Newt Limb Regeneration

**Figure 1:**
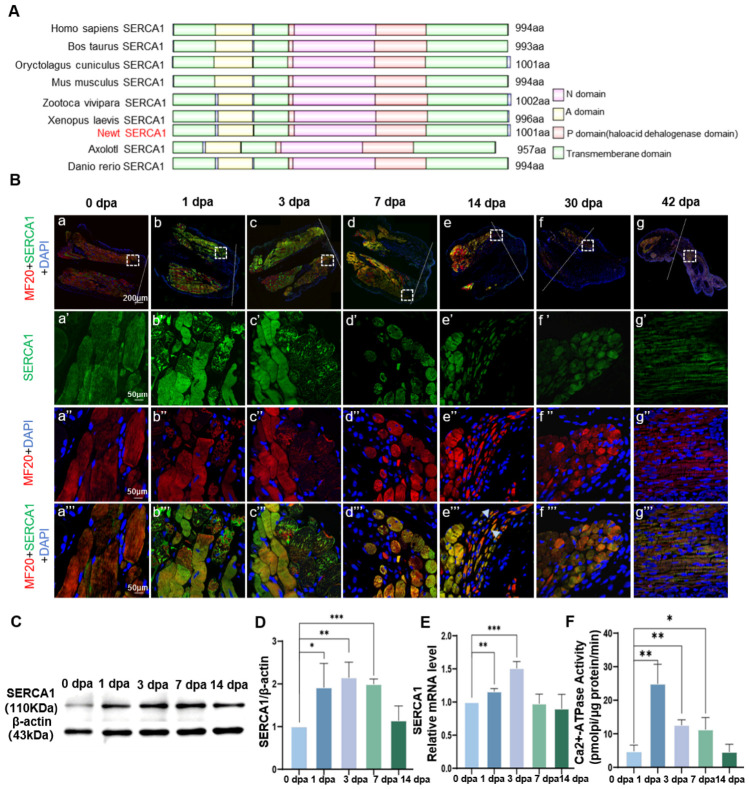
Dynamic expression profile and enzyme activities of SERCA1 during newt limb-regeneration process. (**A**) Sequence alignment of SERCA1 protein (aa, amino acid) across vertebrate species. (**B**) Confocal analysis of colocalization of SERCA1 (green), MF20 (red) and DAPI (blue) in regeneration limb tissue at 0, 1, 3, 7, 14, 30 and 42 dpa. Dashed lines show the limb amputation plane. Illustrations in (**a**–**g**) (dashed boxes) are shown at higher magnification in (**a′**–**g′**), (**a″**–**g″**), and (**a‴**–**g‴**) (*n* = 3). (**C**) Western blotting of SERCA1 protein at 0, 1, 3, 7 and 14 dpa (*n* = 3). (**D**) Statistical analysis of the western blotting images (*n* = 3). (**E**) qRT-PCR of SERCA1 mRNA expression at 0, 1, 3, 7 and 14 dpa (*n* = 3). (**F**) SERCA1 enzyme activity measurement at 0, 1, 3, 7 and 14 dpa (*n* = 3). * *p* < 0.05, ** *p* < 0.01, *** *p* < 0.001. The original Western blot images are summarized in File S1.

SERCA1 is a 110 kDa integral SR membrane Ca^2+^ transporter enzyme and is the major regulator of intracellular Ca^2+^ homeostasis in skeletal muscle cells. We first aligned the amino acid sequences and structural domains of SERCA1 across animal species. The results showed the SERCA1 protein sequences and functional domains were stable and highly homologous among vertebrate taxa, which comprises the actuator (A), phosphorylation (P), nucleotide-binding (N) domains and a transmembrane (M) domain (Figure 1A). To study the role of SERCA1 in initiating the newt limb-regeneration process, the expression profile of SERCA1 from wound healing to blastema proliferation stages was detected. Upon limb amputation, early immediate up-regulation of SERCA1 mRNA and protein expressions were detected at 1 dpa that persisted till 7 dpa, and returned to basal level by 14 dpa (Figure 1C–E). Accordingly, SERCA1 enzyme activity assay also showed a significant increase that peaked at 1 dpa, persisted till 7 dpa and declined to the basal level at 14 dpa (Figure 1F). To observe the spatial-temporal pattern of SERCA1 deposition during the newt limb-regeneration process, regeneration limb tissue was co-stained with SERCA1 and MF20 (a sarcomeric myosin marker) (Figure 1B). Immunofluorescent images showed that SERCA1 deposition was co-localized with MF20 expression in skeletal muscle cells as skeletal muscle cells went through robust fragmentation, dedifferentiation and re-differentiation cellular changes (Figure 1B). In comparison to the elongated, tubular-shaped skeletal muscle cells (Figure 1(Ba‴–Bc‴)), at blastema formation stage (7 dpa), dissociation of muscle fiber bundles and fragmentated muscle cells was observed (Figure 1(Bd‴)). At 14 dpa of blastema proliferation stage, robust dedifferentiated muscle cells marked by MF20 were formed (Figure 1(Be‴), arrowheads). During the pattern formation (30 dpa) and digits formation stages (42 dpa), blastema cells were observed to differentiate and re-develop into mature multinucleated muscle fibers labeled with MF20 and SERCA1 (Figure 1(Bf‴,Bg‴)). 

### 3.2. Knocking Down SERCA1 Expression and Suppressing SERCA1 Activity Disrupted Blastema Formation and Sabotaged Limb Regeneration

To investigate the role of SERCA1 during limb regeneration, a shRNA-SERCA1 plasmid was constructed by engineering shRNA-SERCA1 into the pDsRed-shRNA vector (Supplementary Figure S1A) that was electroporated into the limb tissue 3 days before the limb amputation operation (Supplementary Figure S1B). The shRNA-SERCA1 plasmid contains a red fluorescent element DsRed-express2. We observed that the tissue showed red fluorescent signals at 3 days post-electroporation (Supplementary Figure S1C). qPCR results showed significant increases in DsRed mRNA expression in vector-electroporated limb tissue at 0, 3 and 7 dpa, and became insignificant at 14 and 30 dpa (Supplementary Figure S1D), indicating that the plasmid was successfully transfected into newt limb tissue and lasted for 1 week.

**Figure 2:**
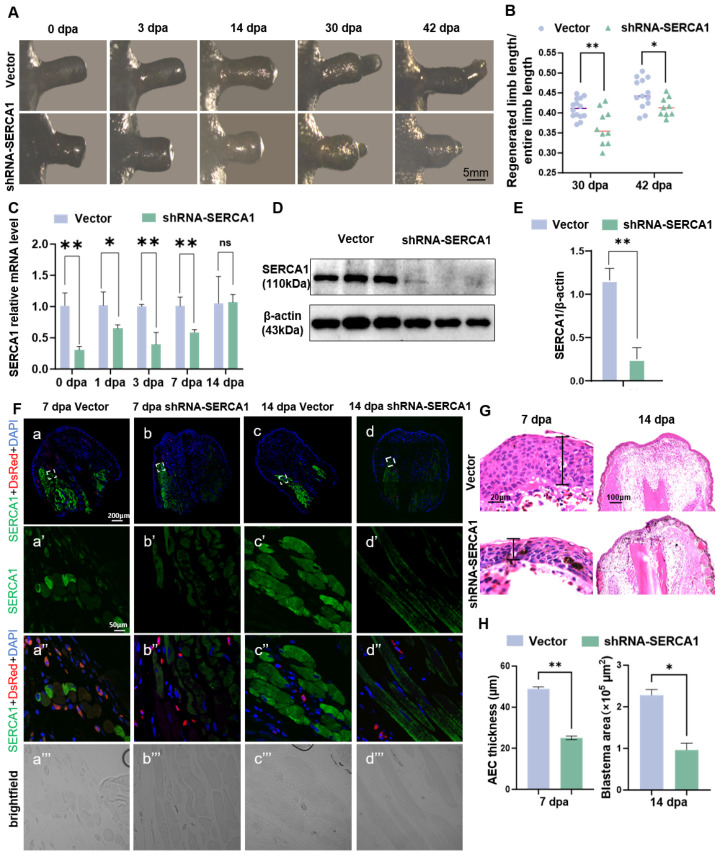

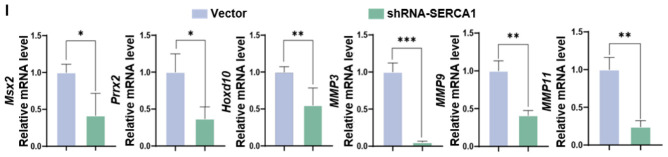
Knocking down SERCA1 expression disrupted the pattern of blastema formation and inhibited limb regeneration. shRNA-SERCA1 plasmid was electroporated into the limb tissue 3 days before limb amputation. (**A**) Images of regenerating newt limbs of control vector and shRNA-treated groups at 0, 3, 14, 30 and 42 dpa. (**B**) Measurement of regenerated tissue length vs. whole limb length in control vector (*n* = 14) and shRNA-treated groups (*n* = 10) at 30 and 42 dpa. (**C**) shRNA-SERCA1 interference efficiency at 0,1,3,7 and 14 dpa (*n* = 3). (**D**) Western blotting of SERCA1 expression in regenerating tissue with shRNA and control vector treatment at 3 dpa. (**E**) Statistical analysis of the western blotting analysis (*n* = 3). (**F**) Confocal analysis of SERCA1 (green), DsRed (red) and DAPI (blue) on regenerating limb tissue sections (7, 14 dpa). shRNA-SERCA1-treated animals showed multinucleated muscle fiber morphology, in contrast to the active myotube fragmentation in control animals. Illustrations in (**a**–**d**) (dashed boxes) are shown at higher magnification in (**a′**–**d′**), (**a″**–**d″**), and (**a‴**–**d‴**) (*n* = 3). (**G**) H&E staining of regenerating limb tissue sections (7, 14 dpa) in control and shRNA-treated newts (*n* = 3). (**H**) Measurement of AEC thickness in control and shRNA-treated newts using ImageJ. (**I)** qRT-PCR examination of mRNA expression profiles of key genes regulating blastema formation in control and shRNA-treated newt limbs at 3 dpa (*n* = 3). ns, not significant (*p* > 0.05), * *p* < 0.05, ** *p* < 0.01, *** *p* < 0.001. The original Western blot images are summarized in File S1.

With shRNA-SERCA1 transfection, newt limb regeneration was markedly delayed. By 42 dpa, the animals showed cone-shaped limb-bud formation, while the control vector treated animals that had progressed with limb regeneration and digits formation (Figure 2A). The regenerated limb length relative to the total limb length was significantly lower in the SERCA1 knocking-down group (Figure 2B). To examine the efficacy of shRNA transfection, qRT-PCR at continuous time points of limb regeneration (0, 1, 3, 7 and 14 dpa) was conducted and showed significantly decreased SERCA1 mRNA expression in regenerating limb tissue with shRNA transfection compared with the control vector group by 7 dpa (Figure 2C). Western blotting analysis of the regenerative tissue demonstrated significantly suppressed SERCA1 protein expression levels in the shRNA-treated group at 3 dpa, which confirmed shRNA-SERCA1 decreased SERCA1 protein expression in vivo (Figure 2D,E). Immunofluorescent staining of SERCA1 showed multinucleated muscle fiber morphology in shRNA-SERCA1-treated animals during blastema formation (7 dpa) and proliferation (14 dpa) stage, in contrast to active myotube fragmentation in regenerating tissue of control animals (Figure 2F). Histological examination showed significantly reduced apical epithelial cap thickness (Figure 2G,H) as well as disrupted blastema formation pattern (14 dpa) (Figure 2G) in shRNA-SERCA1-treated animals. Also, there were misregulated pro-regenerative gene expression profiles in the shRNA-SERCA1-treated group. Expressions of key genes that have been reported to regulate blastema formation were found to be significantly inhibited in the shRNA-SERCA1-treated group, including *Msx2* [30,31], *Prrx2* (Paired Related Homeobox 2) [32] and *Hoxd10* (Homeobox D10) [33], as well as expressions of *MMP3*, *MMP9* and *MMP11* [32,33,34], which play vital roles in ECM remodeling (Figure 2I). 

**Figure 3:**
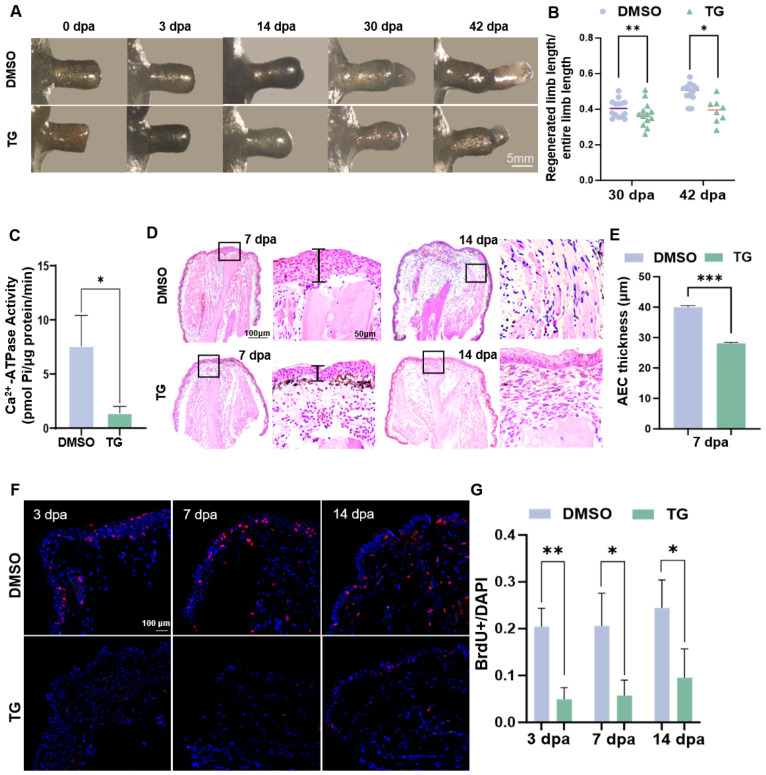
Suppressing SERCA1 activity disrupted the pattern of blastema formation and proliferation, resulting in inhibited limb regeneration. (**A**) Images of regenerating newt limbs in DMSO and Thapsigargin (TG)-treated groups at 0, 3, 14, 30 and 42 dpa. (**B**) Measurement of regenerated tissue length vs. whole limb length in DMSO (*n* = 14) and Thapsigargin-treated (*n* = 13) groups at 30 and 42 dpa. (**C**) SERCA1 enzyme activity assay of regenerating tissue of DMSO or TG-treated newts at 3 dpa (*n* = 3). (**D**) H&E staining of regenerating limb tissue sections, and (**E**) measurement of AEC thickness at 7 dpa in control and Thapsigargin-treated newts (*n* = 3). Bars label AEC structure. (**F**) BrdU (red) and DAPI (blue) staining of regenerating tissue sections and (**G**) quantification of BrdU+ nuclei/total nuclei (3, 7, 14 dpa) (*n* = 3). * *p* < 0.05, ** *p* < 0.01. *** *p* < 0.001.

Also, we treated newts with the pharmaceutical compound Thapsigargin (TG), which is a potent irreversible inhibitor of SERCA1 enzymatic activity [35], by soaking the newts in 0.1 μM TG solution throughout limb regeneration. Consistent with the shRNA-SERCA1 knockdown results, TG-treated newts exhibited markedly delayed limb regeneration compared with the DMSO-treated control group (Figure 3A,B). SERCA1 enzyme activity of regenerating limb tissue was measured at 3 dpa (AEC formation) and was significantly suppressed with TG treatment (Figure 3C). H&E staining showed decreased AEC thickness (Figure 3D,E) and malformation of blastema structure in TG-treated newts (Figure 3D). BrdU analysis indicated that TG treatment suppressed cell proliferation in regenerating limb tissue at 3, 7 and 14 dpa (Figure 3F,G). Although shRNA-SERCA1 transfection persisted for about one week in regenerating limb tissue, the severity of regeneration delay and the phenotype were similar to that of the 6-week TG treatment group, supporting the important role of SERCA1 activity in early limb regeneration stages including AEC formation and blastema formation. Taken together, our data support the involvement of SERCA1 activity in key events of early limb regeneration, including AEC formation, blastema formation, proliferation, and myotube dedifferentiation.

### 3.3. SERCA1 Was Post-Transcriptionally Regulated by miR-20487-5p

**Figure 4:**
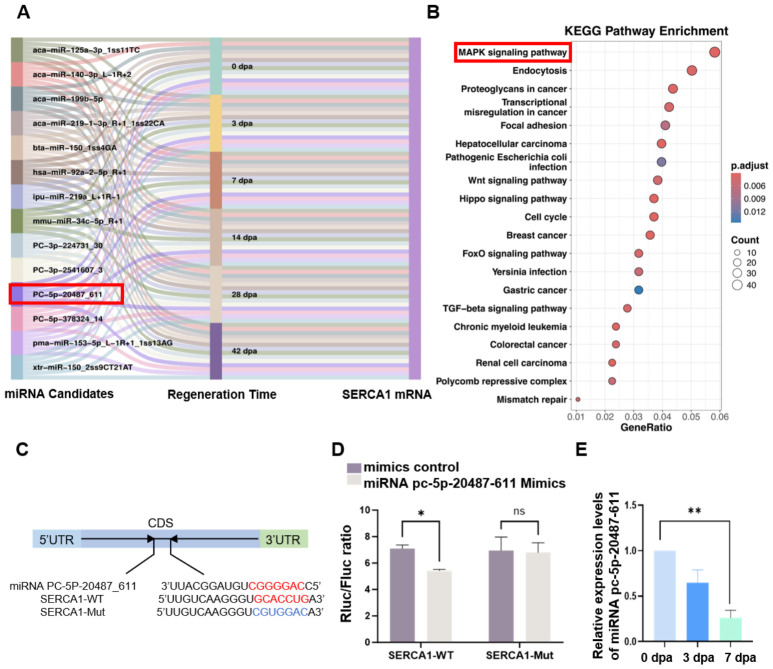
SERCA1 was targeted and negatively regulated by miR-20487-5p. (**A**) An alluvial plot to illustrate the correlation among DE miRNAs, SERCA1 mRNA and regeneration time points. (**B**) KEGG pathway enrichment analysis of the DE miRNA-targeted mRNAs during limb-regeneration process (Benjamini–Hochberg adjusted *p* < 0.05). (**C**) The 3′UTR SERCA1 wild type (WT) matches the seed sequence of miR-20487-5p. (**D**) Dual-luciferase reporter analysis was performed by co-transfecting 293T cells with miR-20487-5p mimics and a luciferase reporter gene ligated to the SERCA1 insertion sequence (*n* = 3). (**E**) qRT-PCR analysis of miR-20487-5p expressions at 0, 3 and 7 dpa (*n* = 3). ns, not significant (*p* > 0.05), * *p* < 0.05, ** *p* < 0.01.

MicroRNAs are reported as dynamic players in timing and organizing gene expression patterns during tissue/organ regeneration processes [36,37,38,39]. Previous studies have identified the microRNAs involved in post-transcriptional modifications of SERCA protein in human and mouse tissues, such as miR-328, miR-25, miR-708, miR-330-5p, miRNA-30b and miRNA-151-3p [40,41,42,43,44,45,46]. Our work reported an integrative analysis of microRNAome, transcriptome and proteome during the entire newt limb-regeneration process (0–42 dpa) [16]. To identify the epigenetic regulatory factor of SERCA1 during newt limb-regeneration, the microRNAome and transcriptome data from the previous work were analyzed. By using the miRanda (v3.3a) and TargetScan (Release 8.0) software, 14 DE miRNAs that were predicted to bind with the CDS region of SERCA1 mRNA were identified, which were negatively correlated with SERCA1 mRNA expression across newt limb-regeneration time points, as shown by the alluvial plot (Figure 4A). Among these miRNAs, the miR-20487-5p (5′-CCAGGGGCUGUAGGCAUU-3′) exhibited the highest binding score with SERCA1 mRNA (Supplementary Figure S2). KEGG enrichment analysis of the DE miRNA-targeted mRNAs highlighted involvement of several signaling pathways including the MAPK, Hippo and Wnt signaling pathways (Figure 4B). Then, dual-luciferase reporter assay was performed to assess the regulatory effect of miR-20487-5p on SERCA1. Results showed the luciferase activity of WT SERCA1 3′UTR in the 293T cells co-transfected with miR-20487-5p mimics was significantly decreased, while that of the mutant SERCA1 3′UTR treatment did not change (Figure 4D). The data suggested miR-20487-5p effectively suppressed SERCA1 expression in vitro. qRT-PCR results showed a significant decrease in miR-20487-5p expression at the blastema formation stage, reciprocal to the SERCA1 expression pattern (Figure 4E).

### 3.4. Exogenous miR-20487-5p Agomir Suppressed SERCA1 Expression and Delayed Limb Regeneration

**Figure 5:**
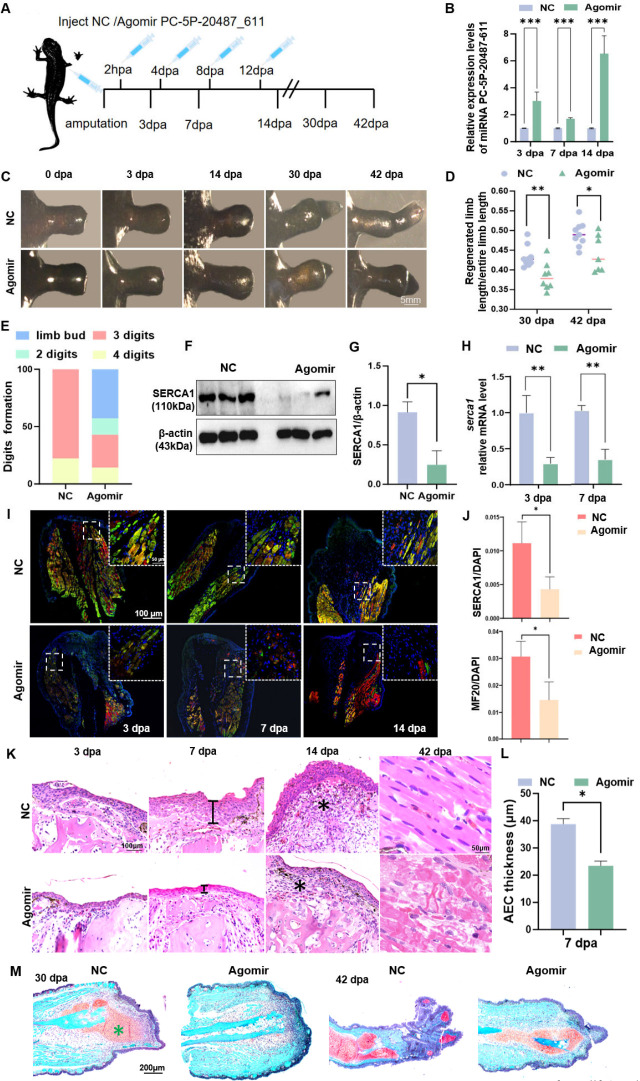
Exogenous administration of miR-20487-5p Agomir suppressed SERCA1 expression and interrupted limb-regeneration process. (**A**) Schematic diagram of the miR-20487-5p Agomir experiment. (**B**) qRT-PCR analysis of miR-20487-5p level in regenerating tissue at 3, 7 and 14 dpa (*n* = 3). (**C**) Representative images of newt limb-regeneration process in NC and Agomir-treated groups at 0, 3, 14, 30 and 42 dpa. (**D**) Measurement of the ratio of regenerated tissue length to whole limb length in NC (*n* = 9) and Agomir-treated (*n* = 8) groups at 30 and 42 dpa. (**E**) Quantification of digit formation in NC (*n* = 9) and Agomir-treated (*n* = 7) groups at 42 dpa. (**F**) Western blotting analysis of SERCA1 expression at 3 dpa with miR-20487-5p Agomir and NC treatment (*n* = 5). (**G**) Statistical analysis of the western blotting assay. (**H**) qRT-PCR assessment of SERCA1 mRNA expression at 3 and 7 dpa with miR-20487-5p Agomir and NC treatment (*n* = 5). (**I**) Confocal analysis of SERCA1 (Alexa Fluor 488, green), MF20 (Alexa Fluor 594, red) and DAPI (blue) in regenerating limb tissue at 3, 7 and 14 dpa with miR-20487-5p Agomir and NC treatment (*n* = 3). Yellow color shows the colocalizations of SERCA1 and MF20. (**J**) Quantification of SERCA1/DAPI and MF20/DAPI positive cell ratios in miR-20487-5p Agomir- and NC-treated limb tissue at 14 dpa. (**K**) Representative images of HE-stained regenerating limb tissue sections of NC or miR-20487-5p Agomir-treated groups (*n* = 3). Bars label AEC structure and asterisks label blastema. (**L**) Measurement of AEC thickness in NC or miR-20487-5p Agomir-treated newts (*n* = 3). (**M**) Safranin O-Fast Green staining of regenerating limb tissue sections at 30 and 42 dpa (*n* = 3). The asterisk indicates cartilage regeneration in NC-treated animals at 30 dpa. * *p* < 0.05, ** *p* < 0.01, *** *p* < 0.001. The original Western blot images are summarized in File S1.

To assess the regulatory role of miR-20487-5p on SERCA1 in vivo, a chemically synthesized mature form of miR-20487-5p Agomir was synthesized and administered intramuscularly at the midstylopod level of the forelimb at 4-day intervals (0, 4, 8, 12 dpa) (Figure 5A). qRT-PCR results showed significantly increased miR-20487-5p levels in regenerating tissue (3, 7 and 14 dpa) of miR-20487-5p Agomir-treated limb tissue (Figure 5B). With exogenous miR-20487-5p Agomir administration, the newt limb regeneration was severely delayed at 42 dpa (Figure 5C). The regenerated tissue length was significantly shorter and digits formation was disrupted in miR-20487-5p Agomir-treated animals (Figure 5D,E). qRT-PCR and eestern blotting analysis of the regenerative tissue demonstrated significantly suppressed SERCA1 mRNA and protein expression levels in the Agomir-treated group, which confirmed miR-20487-5p targeted and negatively regulated SERCA1 expression in vivo (Figure 5F,G,H). Immunofluorescence staining of SERCA1 and MF20 of regenerating tissue sections showed suppressed skeletal muscle cell dedifferentiation in Agomir treatment animals (Figure 5I). Moreover, the ratios of SERCA1-positive and MF20-positive cells to total cell number in regenerating tissue were significantly decreased in the Agomir-treated group (Figure 5J). H&E staining of tissue sections showed disruptions of AEC formation, blastema formation and skeletal muscle fiber disorganization during the tissue-regeneration process in Agomir-treated animals (Figure 5K,L, Supplementary Figure S3). Moreover, the Safranin O-Fast Green staining of limb tissue at 30 dpa and 42 dpa was performed to examine limb bone and joint regeneration, which showed a severely disrupted cartilage and skeletal regeneration pattern in Agomir-treated animals in contrast to well-regenerated elbow and finger digit skeletal structure observed in the control group, suggesting inhibiting SERCA1 expression affected limb skeletal regeneration at the pattern and digits formation stages (Figure 5M).

### 3.5. miR-20487-5p/SERCA1 Perturbation Reduced MAPK/ERK Activation During Newt Limb Regeneration

**Figure 6:**
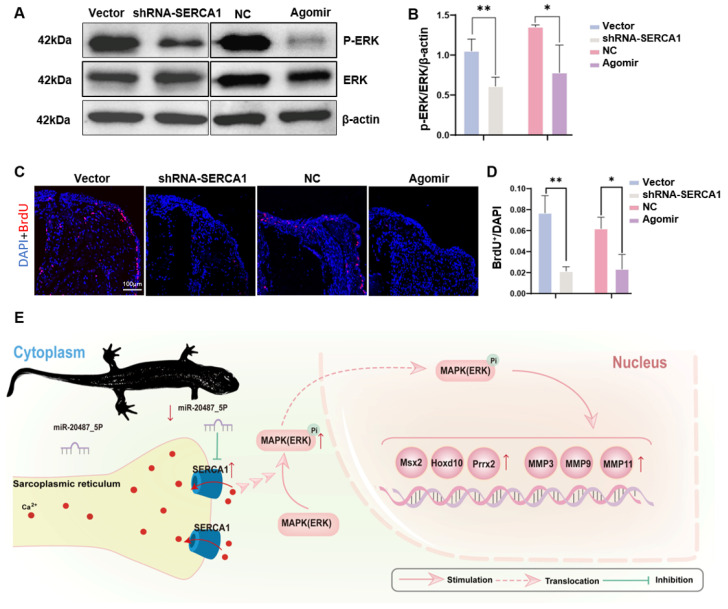
SERCA1/miR-20487-5p perturbation reduced MAPK/ERK activation during newt limb regeneration. (**A**) Western blotting and (**B**) quantification of total ERK and phosphorylated ERK in regenerating limb tissue that were shRNA-treated or Agomir-treated at 3 dpa (*n* = 3). (**C**) BrdU staining (red) and (**D**) quantification of the BrdU+ nuclei/total nuclei (DAPI, blue) in shRNA-treated or Agomir-treated regenerating newt limbs at 3 dpa (*n* = 3). (**E**) Schematic diagram showing the activation of the miR-20487-5p/SERCA1 axis and the potential involvement of its downstream MAPK/ERK activation in newt limb regeneration after injury. * *p* < 0.05, ** *p* < 0.01. The original Western blot images are summarized in File S1.

We further investigated signaling changes associated with the pro-regenerative effects of SERCA1. KEGG analysis of the microRNA transcriptomic data showed enrichment of MAPK/ERK signaling during newt limb-regeneration (Figure 4B). Extensive studies have demonstrated important roles of MAPK/ERK signaling in organ regeneration across multiple model systems [47]. Therefore, we examined ERK phosphorylation after suppressing SERCA1 expression with shRNA-SERCA1 or increasing miR-20487-5p levels with miR-20487-5p Agomir in the newt limb-amputation model. Western blotting analysis showed a significant reduction in phosphorylated ERK protein expression in either the shRNA-treated or Agomir-treated group at 3 dpa, suggesting that ERK activation was reduced following perturbation of the miR-20487-5p/SERCA1 axis (Figure 6A,B). Because MAPK/ERK activation is known to promote cell proliferation, we also examined BrdU incorporation. BrdU analysis showed significantly suppressed cell proliferation in shRNA-SERCA1 or miR-20487-5p Agomir-treated animals at 3 dpa. Therefore, we speculate that MAPK/ERK signaling may participate in the effects associated with miR-20487-5p/SERCA1 activity during limb regeneration initiation (Figure 6C,D).

In conclusion, our results showed that the miR-20487-5p/SERCA1 axis was altered during newt limb regeneration and this affected MAPK/ERK activation, which may participate in early regenerative events such as skeletal myotube dedifferentiation, ECM remodeling and blastema formation (Figure 6E). Our data support the involvement of the miR-20487-5p/SERCA1 axis in early newt limb regeneration and suggest an association between this axis and ERK activation.

## 4. Discussion

Regeneration initiation involves a complex series of cellular and biological processes. The Ca^2+^ signaling is among the earliest responsive signaling mechanisms to detect injury stimuli and subsequently triggers highly dynamic downstream signaling events. Franklin et al. investigated roles of multiple ion channels including the calcium channel during axolotl tail regeneration, which showed that pharmaceutical calcium ion channel antagonists suppressed cell proliferation and completely inhibited axolotl tail regeneration [47]. The present study aimed at investigating the role of the major intracellular calcium homeostasis regulator, SERCA1 pump, in initiating the newt limb-regeneration response. 

A tightly controlled regulation of calcium signaling is essential for cellular functions. Studies have reported that the clearance of free intracellular Ca^2+^, as well as refill of Ca^2+^ storage upon reception of stimuli, generate strong signaling responses that affect cell fate. In human myoblast differentiation, ER stress activates STIM1 (Stromal Interaction Molecule 1) to trigger store-operated calcium entry to increase ER Ca^2+^ levels and balance proteostasis [48]. During C2C12 mouse skeletal-muscle cell differentiation, silencing SERCA1b causes altered calcium flux that suppresses cell proliferation and decreases myotube nuclear numbers [49]. Here, a rapid elevation of SERCA1 mRNA expression, protein expression and enzyme activity during early limb regeneration was observed. Our confocal analysis showed that SERCA1 expression was enriched in fast-twitch skeletal muscle and was present in the entire process of newt skeletal-muscle regeneration, including fragmentation of skeletal-muscle fibers, proliferation and re-development into skeletal muscle. In the newt limb-amputation model, suppression of SERCA1 expression by shRNA or irreversible inactivation of the SERCA1 pump with TG disrupted blastema formation and resulted in severely impaired newt limb regeneration, suggesting SERCA1 was required in newt limb regeneration. 

Intriguingly, SERCA1 and its regulated calcium signaling may be involved in inducing programmed cell death (PCD) response, as demonstrated by one of the machineries initiating skeletal-muscle cell dedifferentiation and regeneration [15]. Wang et al. conducted in vitro experiments using newt A1 cell lines and proved that blocking voltage-dependent anionic channel function with 4,4′diisothicyanatostilbene-2,29-disulfonic-acid (DIDS) prevents myotube fragmentation through inhibiting the PCD process. Consistently, our in vivo study showed that suppressed SERCA1 expression inhibited skeletal-muscle cell fragmentation and dedifferentiation. Also, another study reported that SERCA1 overexpression resulted in DNA fragmentation and apoptotic response in cultured neonatal rat cardiomyocytes, but not in the adult rat cardiomyocytes [50]. Interestingly, our sequence alignment analysis of SERCA1 across different animal phyla showed that the protein structure is highly conserved among species. However, this high sequence similarity does not give rise to the same biological functions in cells from different species or with different regenerative capacities upon injury. Therefore, how SERCA1 is activated in cells with different regenerative capacities and how it is involved in inducing PCD response during the skeletal-muscle cell regeneration process still require in-depth investigations. 

miRNAs are important regulatory mechanisms to control their target gene expressions. Currently, there is no report on miRNAs targeting SERCA1 mRNA in the newt limb-regeneration process. Specific miRNAs are reported to directly target SERCA2a mRNA in mice and humans, and play functional roles in the progression of cardiac hypertrophy through regulating SERCA2a expression. For instance, intervention against miR-25 in heart-failure mice restores SERCA2a function, alleviates disease progression and improves cardiac function [46]. Suppression of miR-328 overstimulation in cardiac-hypertrophy mice rescues SERCA2a expression, and consequently attenuates cardiac hypertrophy via suppressing calcieurin and NFAT activities [43]. In the present study, miR-20487-5p was identified and further confirmed to target SERCA1 and negatively regulate its expression during the newt limb-regeneration process. Further experiments showed exogenous miR-20487-5p Agomir treatment disrupted AEC and blastema formation and suppressed myotube dedifferentiation that sabotaged limb-regeneration. Our findings suggested miR-20487-5p exhibited functional roles in participating in newt limb regeneration initiation by epigenetically regulating SERCA1. Since we only investigated the miRNA predicted with the highest binding score, it is highly likely that other miRNAs were also involved in regulating SERCA1 expression according to our miRanda and TargetScan prediction analysis. Further systematic investigations are required to identify other miRNAs potentially targeting SERCA1. 

The calcium ion flux and MAPK/ERK signaling have been reported as tightly coordinated signaling mechanisms that regulate tissue regenerative activities [51]. Cellular study shows that SERCA controls G1/S transition in cell-cycle progression through regulating cytosolic calcium fluctuations and the downstream MAPK/ERK phosphorylation [52]. Okuda et al. recently demonstrated that the initial rapid ERK activation requires calcium signaling to facilitate regenerative angiogenesis during zebrafish wound healing [53]. The MAPK/ERK is a highly conserved pathway that stands out as a key player in multiple tissue/organ regeneration processes across different animal species [54]. Activated MAPK/ERK signaling mediates a variety of pro-regenerative cellular responses, such as facilitating cardiomyocytes rearrangement, dedifferentiation and proliferation to reactivate adult mice’s cardiomyocyte-regenerative potential after injury [55]; promoting cell fate turn-over and cell cycle re-entry in salamander skeletal muscle regeneration [14,56]; and choreographing tissue growth and morphogenesis during zebrafish scale regeneration [57]. There is evidence that inhibition of SERCA on ER blocks ERK activation, whereas overexpression of SERCA2 activates the MAPK/ERK signaling pathway and promotes SW480 cell proliferation [58,59]. In agreement with the previous findings, our results highlighted the MAPK/ERK pathway in tissue regeneration process, suggesting that knockdown of SERCA1 significantly suppressed ERK activation and cell proliferation. Moreover, exogenous administration of miR-20487-5p Agomir, which specifically targeted SERCA1, also suppressed ERK activation during the limb-regeneration process. Nevertheless, further study is required to strengthen the link between SERCA1 and ERK signaling and elucidate how MAPK/ERK activity hierarchically affects the regeneration initiation response.

In conclusion, our study explored the significant role of the miR-20487-5p/SERCA1 axis in early newt limb regeneration and its association with MAPK/ERK activation. Our findings suggested that this regulatory axis contributed to myotube dedifferentiation, cell proliferation, ECM remodeling, and blastema formation. Nonetheless, limitations of this work should be acknowledged. First, the functionality of the activated MAPK/ERK signaling during limb regeneration requires further validation. Also, as an extremely complicated biological process, one regulatory axis should not be able to trigger the limb regeneration events. It is highly likely that unexamined signaling downstream of the miR-20487-5p/SERCA1 pathway might participate in mediating its pro-regenerative effects. In addition, more interesting questions need to be addressed, such as elucidating the discrepancies in SERCA1 activity and downstream signaling among species with varying regenerative capacities, and elaborating on the mechanism by which SERCA1 induces myotube fragmentation. Therefore, extensive studies are still required to stitch pieces together to achieve an in-depth understanding of the early cellular and signaling events that successfully initiate the newt limb-regeneration process.

## 5. Conclusions

In this study, we demonstrated early immediate responses of SERCA1 expression and activity during *C. orientalis* limb regeneration. Loss-of-function experiments via shRNA interference and TG treatment using the newt limb-amputation model affected key events including AEC formation, myofiber dedifferentiation and blastema formation, and led to severe limb-regeneration defects. We identified a novel microRNA, miR-20487-5p, that negatively regulated SERCA1 expression during newt limb regeneration. In vivo administration of miR-20487-5p Agomir resulted in regeneration deficiency. The miR-20487-5p/SERCA1 axis was associated with reduced MAPK/ERK activation and may contribute to its pro-regenerative effects. Collectively, our work suggests that the miR-20487-5p/SERCA1 axis, potentially associated with MAPK/ERK activation, is involved in early events of newt limb regeneration. These findings reveal a candidate regulatory axis linking miRNA regulation, calcium transporter activity and MAPK/ERK signaling in newt appendage regeneration, and provide evidence for the translational application of future regenerative medicine research.

## Data Availability

The microRNA sequencing raw data have been deposited to the NCBI Sequence Read Archive (SRA, https://www.ncbi.nlm.nih.gov/sra, accessed on 28 June 2026) with the dataset accession PRJNA1479149.

## Supplementary Materials

The following supporting information can be downloaded at: https://www.mdpi.com/article/10.3390/biology15141107/s1, Table S1: Primers used in this study; Scheme S1: Illustration of tissue sample harvest and in vivo plasmid electroporation experiment procedures. (A) Illustration of regenerating tissue harvest for qRT-PCR/WB/IF. The right forelimb amputations at the midstylopod level on newts were conducted. Tissue samples were harvested 2 mm below the amputation plane at continuous limb regeneration time points. (B) For in vivo plasmid electroporation, plasmid was injected intramuscularly at multiple sites surrounding the midstylopod level of the newt forelimb using a microinjector. Then, electrodes were placed close to the skin without direct contact to avoid skin injury, and the limbs were electroporated using the electroporation equipment; Figure S1: Transfection of pDs-Red shRNA-SERCA1 plasmid into newt limb tissue via electroporation. (A) Modified pDs-Red express2 vector cloned with a U6 promoter and shRNA-SERCA1. (B) pDs-Red shRNA-SERCA1 plasmid was electroporated into newt limb tissue 3 days before limb amputation and regenerating tissue was harvested at 0, 3, 7, 14, 30 dpa. (C) Small piece of muscle tissue was cut off from the electroporated limb at 3 dpa to observe presence of DsRed protein under the fluorescence microscope. (D) qRT-PCR analysis of DsRed mRNA expression in regenerating tissue as illustrated in B (0, 3, 7, 14, 30 dpa) (*n* = 3). * *p* < 0.05, ** *p* < 0.01. *** *p* < 0.001; Figure S2: miR-20487-5p was identified to bind and target SERCA1 mRNA. (A) miR-20487-5p exhibited 2 binding sites with the highest score predicted by the TargetScan software. (B) miRanda software predicted the binding site located in the CDS region of SERCA1 mRNA. (C) the stem-loop structure of miR-20487-5p analyzed by RNAfold software. (D) RNA hybrid software predicted the binding of miR-20487-5p and its targeting SERCA1 nucleotides; Figure S3: HE-staining of regenerating limb tissue in NC and miR-20487-5p Agomir treated animals at continuous time points of limb regeneration process (3, 7, 14, 30, 42 dpa). Arrow heads show AEC structure at 3 and 7 dpa (a′, b′, c′, d′). Asterisks show blastema area at 14 dpa (e′, f′) (*n* = 3); File S1: Original Western Blot images; File S2: Transcriptomic sequencing clean data.

## References

[1] Aztekin C. (2021). Tissues and Cell Types of Appendage Regeneration: A Detailed Look at the Wound Epidermis and Its Specialized Forms. Front. Physiol..

[2] Repesh L.A., Oberpriller J.C. (1978). Scanning electron microscopy of epidermal cell migration in wound healing during limb regeneration in the adult newt, Notophthalmus viridescens. Am. J. Anat..

[3] Kragl M., Knapp D., Nacu E., Khattak S., Maden M., Epperlein H.H., Tanaka E.M. (2009). Cells keep a memory of their tissue origin during axolotl limb regeneration. Nature.

[4] Tanaka H.V., Ng N.C.Y., Yang Yu Z., Casco-Robles M.M., Maruo F., Tsonis P.A., Chiba C. (2016). A developmentally regulated switch from stem cells to dedifferentiation for limb muscle regeneration in newts. Nat. Commun..

[5] McCusker C., Bryant S.V., Gardiner D.M. (2015). The axolotl limb blastema: Cellular and molecular mechanisms driving blastema formation and limb regeneration in tetrapods. Regeneration.

[6] Tanaka E.M. (2016). The Molecular and Cellular Choreography of Appendage Regeneration. Cell.

[7] Sandoval-Guzmán T., Wang H., Khattak S., Schuez M., Roensch K., Nacu E., Tazaki A., Joven A., Tanaka E.M., Simon A. (2014). Fundamental Differences in Dedifferentiation and Stem Cell Recruitment during Skeletal Muscle Regeneration in Two Salamander Species. Cell Stem Cell.

[8] Calve S., Simon H.G. (2011). High resolution three-dimensional imaging: Evidence for cell cycle reentry in regenerating skeletal muscle. Dev. Dyn..

[9] Echeverri K., Clarke J.D., Tanaka E.M. (2001). In vivo imaging indicates muscle fiber dedifferentiation is a major contributor to the regenerating tail blastema. Dev. Biol..

[10] Wang H., Simon A. (2016). Skeletal muscle dedifferentiation during salamander limb regeneration. Curr. Opin. Genet. Dev..

[11] Morrison J.I., Loof S., He P., Simon A. (2006). Salamander limb regeneration involves the activation of a multipotent skeletal muscle satellite cell population. J. Cell Biol..

[12] Sugiura T., Wang H., Barsacchi R., Simon A., Tanaka E.M. (2016). MARCKS-like protein is an initiating molecule in axolotl appendage regeneration. Nature.

[13] Wagner I., Wang H., Weissert P.M., Straube W.L., Shevchenko A., Gentzel M., Brito G., Tazaki A., Oliveira C., Sugiura T. (2017). Serum Proteases Potentiate BMP-Induced Cell Cycle Re-entry of Dedifferentiating Muscle Cells during Newt Limb Regeneration. Dev. Cell.

[14] Walters H.E., Troyanovskiy K.E., Graf A.M., Yun M.H. (2023). Senescent cells enhance newt limb regeneration by promoting muscle dedifferentiation. Aging Cell.

[15] Wang H., Loof S., Borg P., Nader G.A., Blau H.M., Simon A. (2015). Turning terminally differentiated skeletal muscle cells into regenerative progenitors. Nat. Commun..

[16] Yu Y., Tang J., Su J., Cui J., Xie X., Chen F. (2019). Integrative Analysis of MicroRNAome, Transcriptome, and Proteome during the Limb Regeneration of Cynops orientalis. J. Proteome Res..

[17] Chambers P.J., Juracic E.S., Fajardo V.A., Tupling A.R. (2022). Role of SERCA and sarcolipin in adaptive muscle remodeling. Am. J. Physiol. Cell Physiol..

[18] Periasamy M., Kalyanasundaram A. (2007). SERCA pump isoforms: Their role in calcium transport and disease. Muscle Nerve.

[19] Toyoshima C. (2008). Structural aspects of ion pumping by Ca2+-ATPase of sarcoplasmic reticulum. Arch. Biochem. Biophys..

[20] Drachmann N.D., Olesen C., Møller J.V., Guo Z., Nissen P., Bublitz M. (2014). Comparing crystal structures of Ca^2+^-ATPase in the presence of different lipids. FEBS J..

[21] Toyoshima C., Nomura H., Tsuda T. (2004). Lumenal gating mechanism revealed in calcium pump crystal structures with phosphate analogues. Nature.

[22] Atcha H., Jairaman A., Holt J.R., Meli V.S., Nagalla R.R., Veerasubramanian P.K., Brumm K.T., Lim H.E., Othy S., Cahalan M.D. (2021). Mechanically activated ion channel Piezo1 modulates macrophage polarization and stiffness sensing. Nat. Commun..

[23] Zhang J., Li Y., Jiang S., Yu H., An W. (2014). Enhanced endoplasmic reticulum SERCA activity by overexpression of hepatic stimulator substance gene prevents hepatic cells from ER stress-induced apoptosis. Am. J. Physiol. Cell Physiol..

[24] Zheng S., Zhao D., Hou G., Zhao S., Zhang W., Wang X., Li L., Lin L., Tang T.S., Hu Y. (2022). iASPP suppresses Gp78-mediated TMCO1 degradation to maintain Ca(2+) homeostasis and control tumor growth and drug resistance. Proc. Natl. Acad. Sci. USA.

[25] Adams D.S., Masi A., Levin M. (2007). H+ pump-dependent changes in membrane voltage are an early mechanism necessary and sufficient to induce Xenopus tail regeneration. Development.

[26] Perathoner S., Daane J.M., Henrion U., Seebohm G., Higdon C.W., Johnson S.L., Nusslein-Volhard C., Harris M.P. (2014). Bioelectric signaling regulates size in zebrafish fins. PLoS Genet..

[27] Song Y., Li D., Farrelly O., Miles L., Li F., Kim S.E., Lo T.Y., Wang F., Li T., Thompson-Peer K.L. (2019). The Mechanosensitive Ion Channel Piezo Inhibits Axon Regeneration. Neuron.

[28] McLaughlin K.A., Levin M. (2018). Bioelectric signaling in regeneration: Mechanisms of ionic controls of growth and form. Dev. Biol..

[29] Sousounis K., Erdogan B., Levin M., Whited J.L. (2020). Precise control of ion channel and gap junction expression is required for patterning of the regenerating axolotl limb. Int. J. Dev. Biol..

[30] Yilmaz A., Engeler R., Constantinescu S., Kokkaliaris K.D., Dimitrakopoulos C., Schroeder T., Beerenwinkel N., Paro R. (2015). Ectopic expression of Msx2 in mammalian myotubes recapitulates aspects of amphibian muscle dedifferentiation. Stem Cell Res..

[31] Satoh A., Gardiner D.M., Bryant S.V., Endo T. (2007). Nerve-induced ectopic limb blastemas in the Axolotl are equivalent to amputation-induced blastemas. Dev. Biol..

[32] Godwin J.W., Pinto A.R., Rosenthal N.A. (2013). Macrophages are required for adult salamander limb regeneration. Proc. Natl. Acad. Sci. USA.

[33] Haas B.J., Whited J.L. (2017). Advances in Decoding Axolotl Limb Regeneration. Trends Genet..

[34] Vinarsky V., Atkinson D.L., Stevenson T.J., Keating M.T., Odelberg S.J. (2005). Normal newt limb regeneration requires matrix metalloproteinase function. Dev. Biol..

[35] Moller J.V., Olesen C., Picard M., Morth P., Winter A.M.L., Nissen P., Christensen S.B., Sohoel H. (2008). How the sarcoplasmic reticulum Ca2+-ATPase pumps Ca2+ and is inhibited by thapsigargin. J. Gen. Physiol..

[36] Yin V.P., Poss K.D. (2008). New regulators of vertebrate appendage regeneration. Curr. Opin. Genet. Dev..

[37] Witman N., Heigwer J., Thaler B., Lui W.O., Morrison J.I. (2013). miR-128 regulates non-myocyte hyperplasia, deposition of extracellular matrix and Islet1 expression during newt cardiac regeneration. Dev. Biol..

[38] Subramanian E., Elewa A., Brito G., Kumar A., Segerstolpe Å., Karampelias C., Björklund Å., Sandberg R., Echeverri K., Lui W.O. (2023). A small noncoding RNA links ribosome recovery and translation control to dedifferentiation during salamander limb regeneration. Dev. Cell.

[39] Abo-Al-Ela H.G., Burgos-Aceves M.A. (2021). Exploring the role of microRNAs in axolotl regeneration. J. Cell. Physiol..

[40] Stammers A.N., Susser S.E., Hamm N.C., Hlynsky M.W., Kimber D.E., Kehler D.S., Duhamel T.A. (2015). The regulation of sarco(endo)plasmic reticulum calcium-ATPases (SERCA). Can. J. Physiol. Pharmacol..

[41] Ryu S., McDonnell K., Choi H., Gao D., Hahn M., Joshi N., Park S.M., Catena R., Do Y., Brazin J. (2013). Suppression of miRNA-708 by polycomb group promotes metastases by calcium-induced cell migration. Cancer Cell.

[42] Han D., Wang Y., Wang Y., Dai X., Zhou T., Chen J., Tao B., Zhang J., Cao F. (2020). The Tumor-Suppressive Human Circular RNA CircITCH Sponges miR-330-5p to Ameliorate Doxorubicin-Induced Cardiotoxicity Through Upregulating SIRT6, Survivin, and SERCA2a. Circ. Res..

[43] Li C., Li X., Gao X., Zhang R., Zhang Y., Liang H., Xu C., Du W., Zhang Y., Liu X. (2014). MicroRNA-328 as a regulator of cardiac hypertrophy. Int. J. Cardiol..

[44] Wei H., Li Z., Wang X., Wang J., Pang W., Yang G., Shen Q.W. (2015). microRNA-151-3p regulates slow muscle gene expression by targeting ATP2a2 in skeletal muscle cells. J. Cell. Physiol..

[45] Dai L.L., Li S.D., Ma Y.C., Tang J.R., Lv J.Y., Zhang Y.Q., Miao Y.L., Ma Y.Q., Li C.M., Chu Y.Y. (2019). MicroRNA-30b regulates insulin sensitivity by targeting SERCA2b in non-alcoholic fatty liver disease. Liver Int..

[46] Wahlquist C., Jeong D., Rojas-Muñoz A., Kho C., Lee A., Mitsuyama S., van Mil A., Park W.J., Sluijter J.P., Doevendans P.A. (2014). Inhibition of miR-25 improves cardiac contractility in the failing heart. Nature.

[47] Franklin B.M., Voss S.R., Osborn J.L. (2017). Ion channel signaling influences cellular proliferation and phagocyte activity during axolotl tail regeneration. Mech. Dev..

[48] Carreras-Sureda A., Zhang X., Laubry L., Brunetti J., Koenig S., Wang X.X., Castelbou C., Hetz C., Liu Y., Frieden M. (2023). The ER stress sensor IRE1 interacts with STIM1 to promote store-operated calcium entry, T cell activation, and muscular differentiation. Cell Rep..

[49] Tóth A., Fodor J., Vincze J., Oláh T., Juhász T., Zákány R., Csernoch L., Zádor E. (2015). The Effect of SERCA1b Silencing on the Differentiation and Calcium Homeostasis of C2C12 Skeletal Muscle Cells. PLoS ONE.

[50] Wu G., Long X., Marín-García J. (2004). Adenoviral SERCA1 overexpression triggers an apoptotic response in cultured neonatal but not in adult rat cardiomyocytes. Mol. Cell. Biochem..

[51] Ghilardi S.J., O’Reilly B.M., Sgro A.E. (2020). Intracellular signaling dynamics and their role in coordinating tissue repair. Wiley Interdiscip. Rev. Syst. Biol. Med..

[52] Simon V.R., Moran M.F. (2001). SERCA activity is required for timely progression through G1/S. Cell Prolif..

[53] Okuda K.S., Keyser M.S., Gurevich D.B., Sturtzel C., Mason E.A., Paterson S., Chen H., Scott M., Condon N.D., Martin P. (2021). Live-imaging of endothelial Erk activity reveals dynamic and sequential signalling events during regenerative angiogenesis. Elife.

[54] Wen X.M., Jiao L.D., Tan H. (2022). MAPK/ERK Pathway as a Central Regulator in Vertebrate Organ Regeneration. Int. J. Mol. Sci..

[55] Aharonov A., Shakked A., Umansky K.B., Savidor A., Genzelinakh A., Kain D., Lendengolts D., Revach O.Y., Morikawa Y., Dong J. (2020). ERBB2 drives YAP activation and EMT-like processes during cardiac regeneration. Nat. Cell Biol..

[56] Yun M.H., Gates P.B., Brockes J.P. (2014). Sustained ERK activation underlies reprogramming in regeneration-competent salamander cells and distinguishes them from their mammalian counterparts. Stem Cell Rep..

[57] De Simone A., Evanitsky M.N., Hayden L., Cox B.D., Wang J., Tornini V.A., Ou J., Chao A., Poss K.D., Di Talia S. (2021). Control of osteoblast regeneration by a train of Erk activity waves. Nature.

[58] Fan L., Li A., Li W., Cai P., Yang B., Zhang M., Gu Y., Shu Y., Sun Y., Shen Y. (2014). Novel role of Sarco/endoplasmic reticulum calcium ATPase 2 in development of colorectal cancer and its regulation by F36, a curcumin analog. Biomed. Pharmacother..

[59] Fang X., Ni K., Guo J., Li Y., Zhou Y., Sheng H., Bu B., Luo M., Ouyang M., Deng L. (2022). FRET Visualization of Cyclic Stretch-Activated ERK via Calcium Channels Mechanosensation While Not Integrin β1 in Airway Smooth Muscle Cells. Front. Cell Dev. Biol..

