# Supplementary material for: miR-20487-5p/SERCA1/MAPK/ERK Pathway Regulates Newt Limb Regeneration
Source: Biology (Basel). 2026 Jul 9;15(14):1107. doi: 10.3390/biology15141107 (PMC13404080; doi:10.3390/biology15141107)
Supplement: Supplementary file 1 [file biology-15-01107-s001.zip › biology-4387775-Supplementary Materials.pdf]

Supplemental Table S1. Primers used in this study

Primer name	Sequence (5′-3′)	Purpose
SERCA1-QF	GAAAGACCTTGTGGGAGC	qRT-PCR
SERCA1-QR	ATGGACTGGTCAATACGC	qRT-PCR
GAPDH-QF	CTGTCCATGCTGTGACTGCT	qRT-PCR
GAPDH-QR	CACATTGGCGACAGGTACAC	qRT-PCR
MMP3-QF	GATGTTGATGGCATTCAAGCC	qRT-PCR
MMP3-QR	AGTTGGGTCACAGATGGATGG	qRT-PCR
MMP9-QF	CATCGTAGGATTCACCATCG	qRT-PCR
MMP9-QR	ACCACGACCGACTATGACAA	qRT-PCR
MMP11-QF	TCCTTGGCAGCTGAGTAAAG	qRT-PCR
MMP11-QR	CCTCGTGCACTTCGGTAAA	qRT-PCR
Prrx2-QF	GATAGGGAACCTGAGGGAAATG	qRT-PCR
Prrx2-QR	CCTCAGTTCTCGATTGCTCTAAA	qRT-PCR
Hoxd10-QF	CCATCCCTCTCCCTCAAATTC	qRT-PCR
Hoxd10-QR	GCTGATCAAGGAGTCCACTAAA	qRT-PCR
Msx2F	CACCCCTTTCACCACCTCCC	qRT-PCR
Msx2R	AAGGGTAAACTGAAGCCAGG	qRT-PCR
miRNA PC-5P-20487 Primer	CGCCAGGGGCTGTAGGCATT	qRT-PCR
U6-F	CTCGCTTCGGCAGCACA	qRT-PCR
U6-R	AACGCTTCACGAATTTGCGT	qRT-PCR
DsRed-F	CCCGTAATGCAGAAGAAGAC	qRT-PCR
DsRed-R	CGTTGTGGGAGGTGATGT	qRT-PCR
shRNA-SERCA1-F	GATCGGATAAAGTTGACGGAGATGTTTCAAGAGAACATCTCCGTCAACTTTATCCTTTTT	shRNA-SERCA1 vector
shRNA-SERCA1-R	AATTAAAAAGGATAAAGTTGACGGAGATGTTCTCTTGAAACATCTCCGTCAACTTTATCC	shRNA-SERCA1 vector
WT-SERCA1-F	CCGCTCGAGCCAATGCCTGCAACTCT	Cloned into psiCHECK-2 vector
WT-SERCA1-R	AAAGCGGCCGCACCCCACTCTTTGATAACG	Cloned into psiCHECK-2 vector
Mut-SERCA1-F	gacAGGGTGTCATTGATCGTTG	Site-directed mutagenesis cloned into psiCHECK-2 vector
Mut-SERCA1-R	cacgACCCTTGACAAACATCTTG	Site-directed mutagenesis cloned into psiCHECK-2 vector

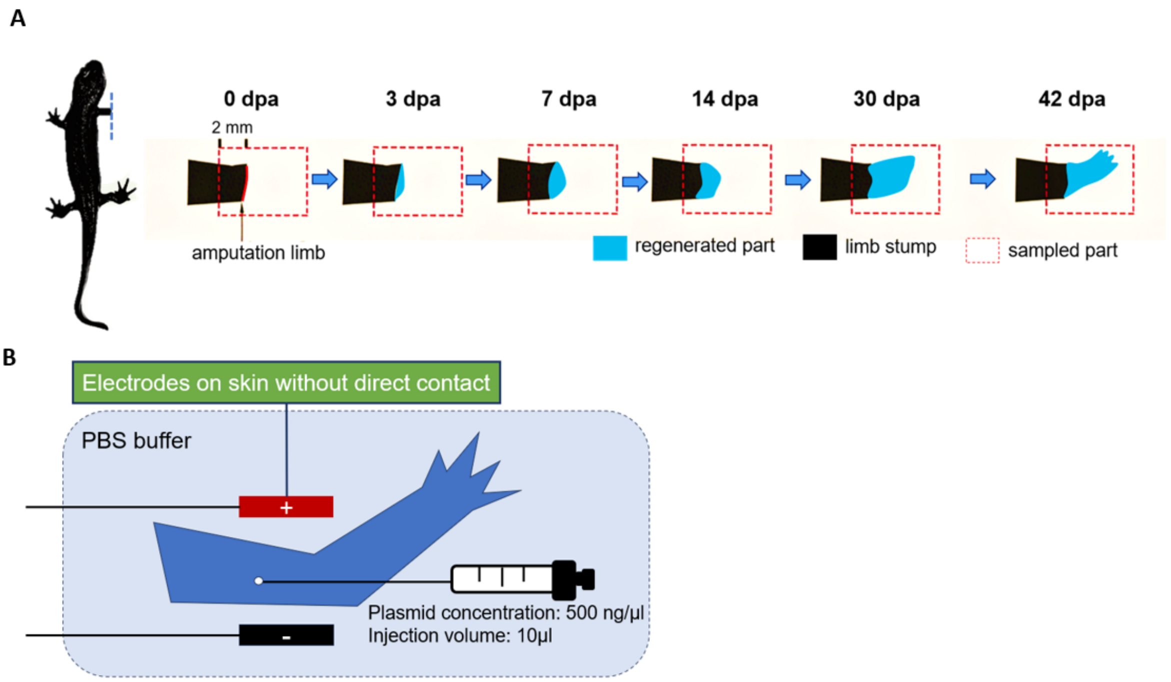

Supplemental Scheme S1. Illustration of tissue sample harvest and in vivo plasmid electroporation experiment procedures. (**A**) Illustration of regenerating tissue harvest for qRT-PCR/WB/IF. The right forelimb amputations at the midstylopod level on newts were conducted. Tissue samples were harvested 2mm below the amputation plane at continuous limb regeneration time points. (**B**) For in vivo plasmid electroporation, plasmid was injected intramuscularly at multiple sites surrounding the midstylopod level of the newt forelimb using a microinjector. Then, electrodes were placed close to the skin without direct contact to avoid skin injury, and the limbs were electroporated using the electroporation equipment.

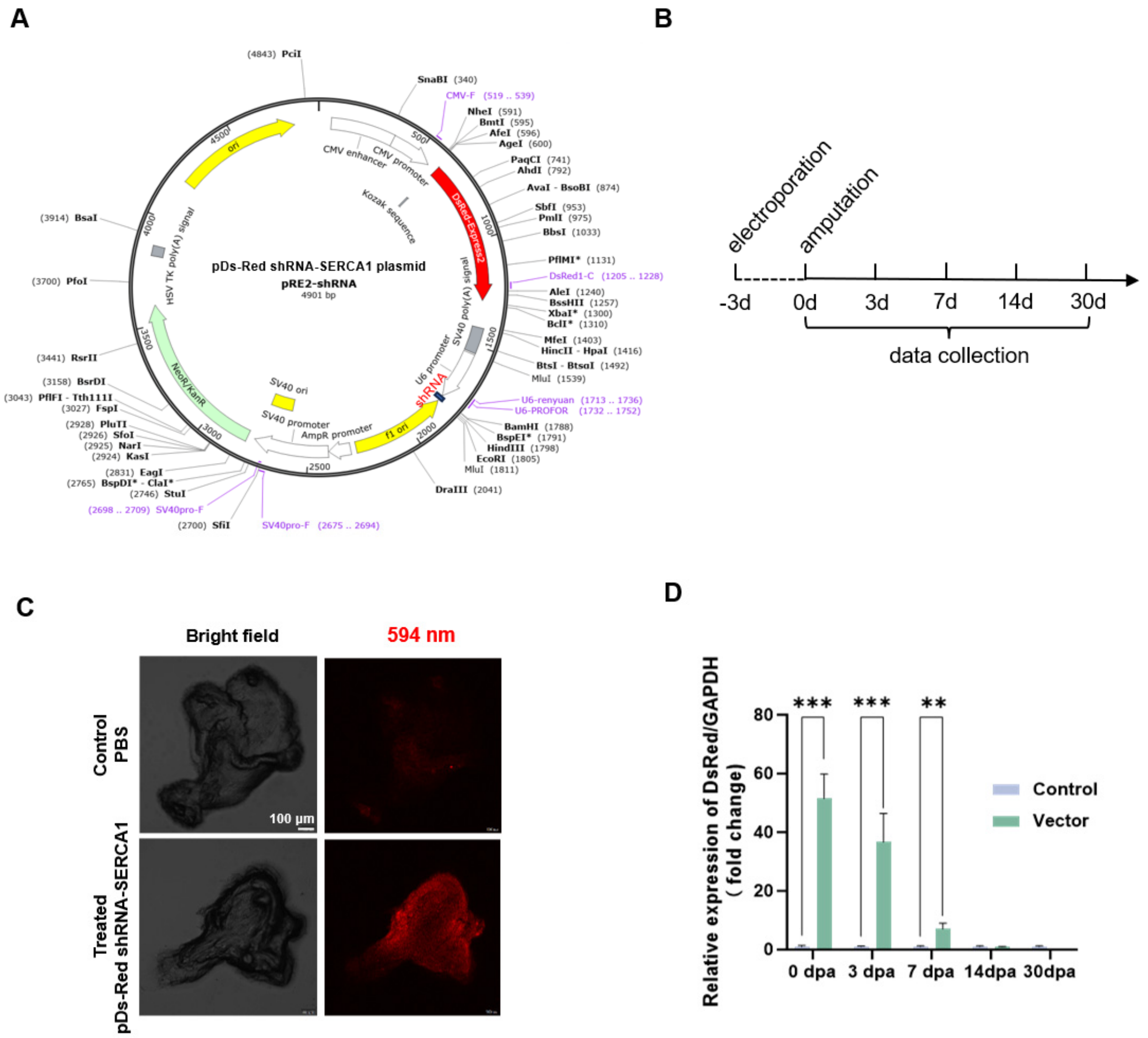

Supplemental Figure S1. Transfection of pDs-Red shRNA-SERCA1 plasmid into newt limb tissue via electroporation. (**A**) Modified pDs-Red express2 vector cloned with a U6 promoter and shRNA-SERCA1. (**B**) pDs-Red shRNA-SERCA1 plasmid was electroporated into newt limb tissue 3 days before limb amputation and regenerating tissue was harvested at 0,3,7,14,30 dpa. (**C**) Small piece of muscle tissue was cut off from the electroporated limb at 3 dpa to observe presence of DsRed protein under the fluorescence microscope. (**D**) qRT-PCR analysis of DsRed mRNA expression in regenerating tissue as illustrated in B (0,3,7,14,30 dpa) (n=3). *P<0.05, **P<0.01. ***P<0.001

A

miRNA ID	Symbol	TargetScan_score	miranda_Energy
hsa-miR-92a-2-5p_R+1	ATP2A1	66	-22.02
hsa-miR-92a-2-5p_R+1	ATP2A1	76	-22.02
hsa-miR-92a-2-5p_R+1	ATP2A1	76	-22.02
PC-5p-20487 611	ATP2A1	60	-19.51
PC-5p-20487 611	ATP2A1	95	-16.49

B

```
Performing Scan: PC-5p-20487_611 vs ATP2A1

Score: 143.000000  Q:2 to 17  R:180 to 199 Align Len (17) (64.71%) (76.47%)
   Forward:

   Query:     3' ttACGGATGT--CGGGGACc 5'
                   ||:| | :  |||||||
   Ref:       5' ttTGTCAAGGGTGCCCCTGa 3'

   Energy:  -16.490000 kCal/Mol
```

C

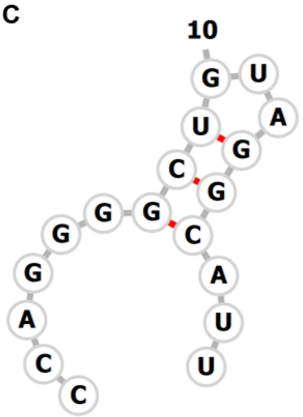

D

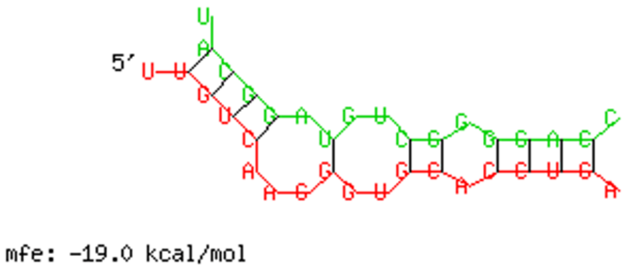

Supplemental Figure S2. miR-20487-5p was identified to bind and target SERCA1 mRNA. (**A**) miR-20487-5p exhibited 2 binding sites with the highest score predicted by the TargetScan software. (**B**) miRanda software predicted the binding site located in the CDS region of SERCA1 mRNA. (**C**) the stem-loop structure of miR-20487-5p analyzed by RNAfold software. (**D**) RNA hybrid software predicted the binding of miR-20487-5p and its targeting SERCA1 nucleotides.

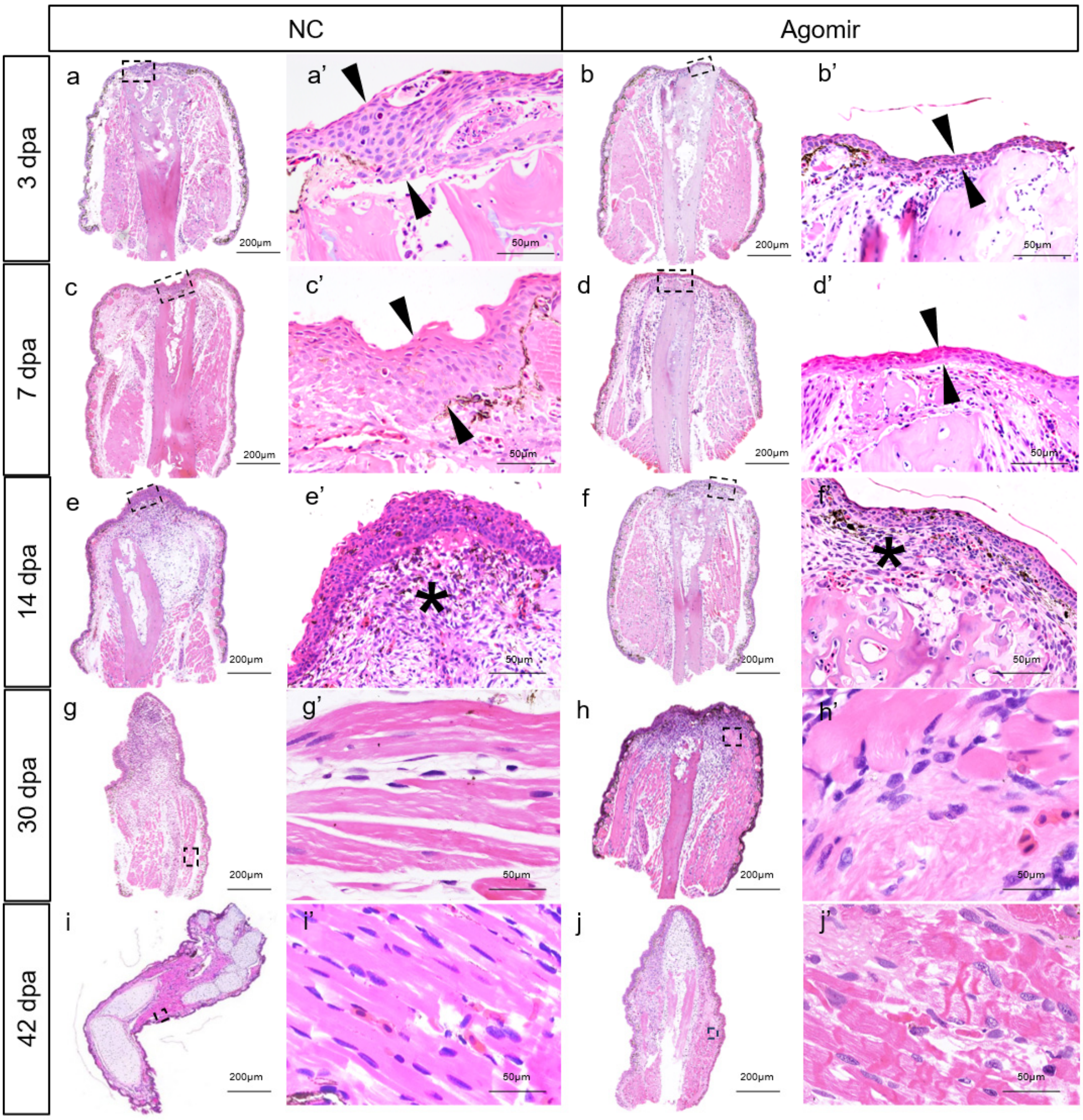

Supplemental Figure S3. HE-staining of regenerating limb tissue in NC and miR-20487-5p Agomir treated animals at continuous time points of limb regeneration process (3, 7, 14, 30, 42 dpa). Arrow heads show AEC structure at 3 and 7 dpa (a′, b′, c′, d′). Asterisks show blastema area at 14 dpa (e′, f′) (*n*=3).
