# Supplementary material for: miR-20487-5p/SERCA1/MAPK/ERK Pathway Regulates Newt Limb Regeneration
Source: Biology (Basel). 2026 Jul 9;15(14):1107. doi: 10.3390/biology15141107 (PMC13404080; doi:10.3390/biology15141107)
Supplement: Supplementary file 1 [file biology-15-01107-s001.zip › File S1.pdf]

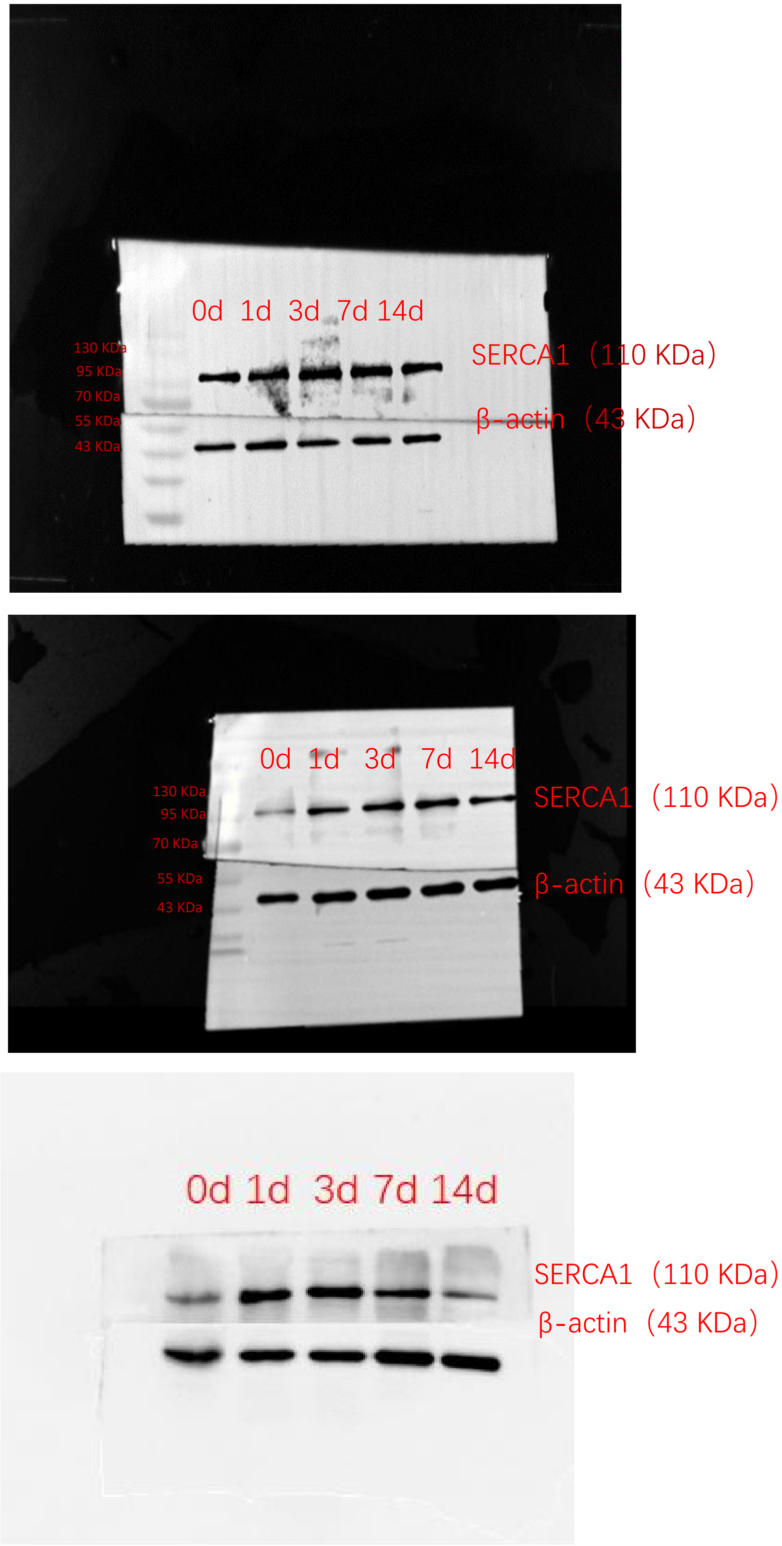

Western blot analysis of SERCA1 protein at the early stage of limb regeneration.

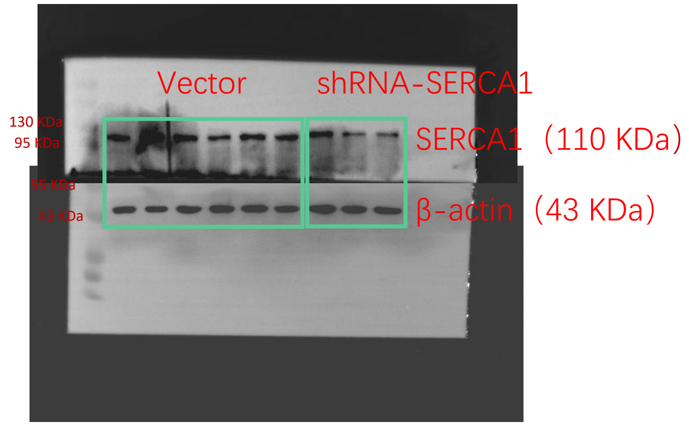

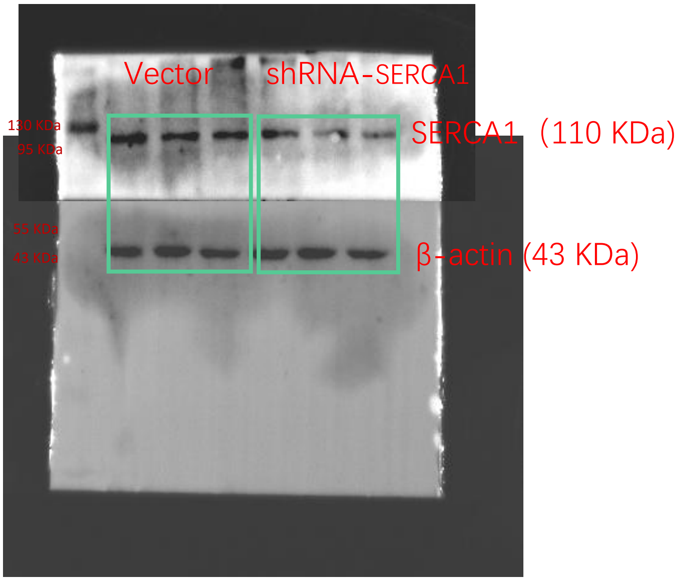

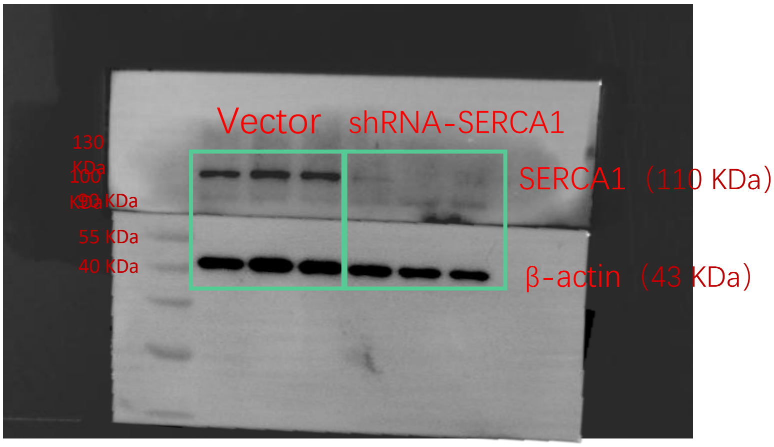

Western blotting of SERCA1 expression at 3 dpa with shRNA-treated.

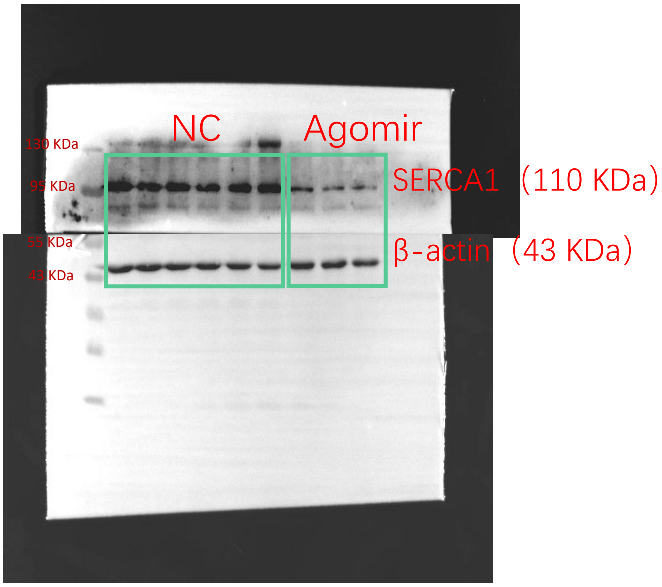

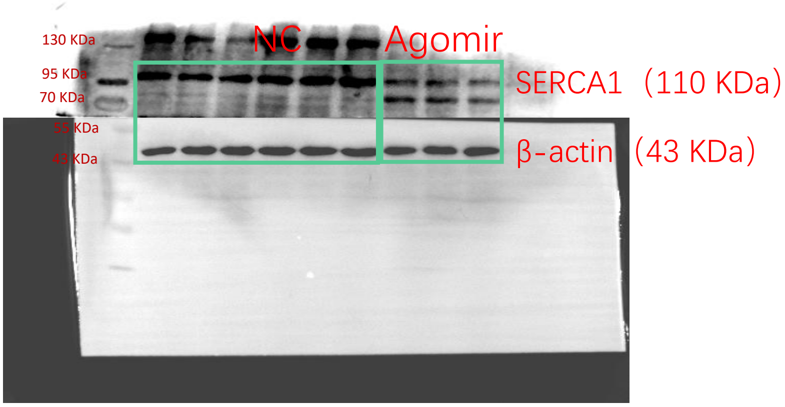

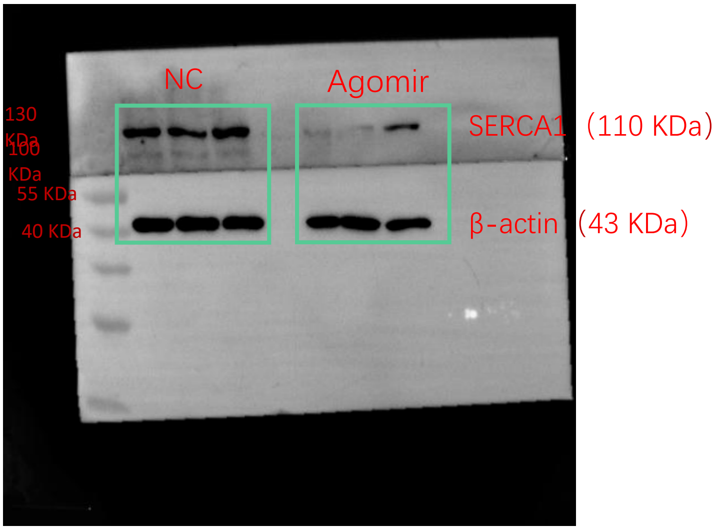

Western blotting of SERCA1 expression at 3 dpa with miR-20487-5p Agomir treatment.

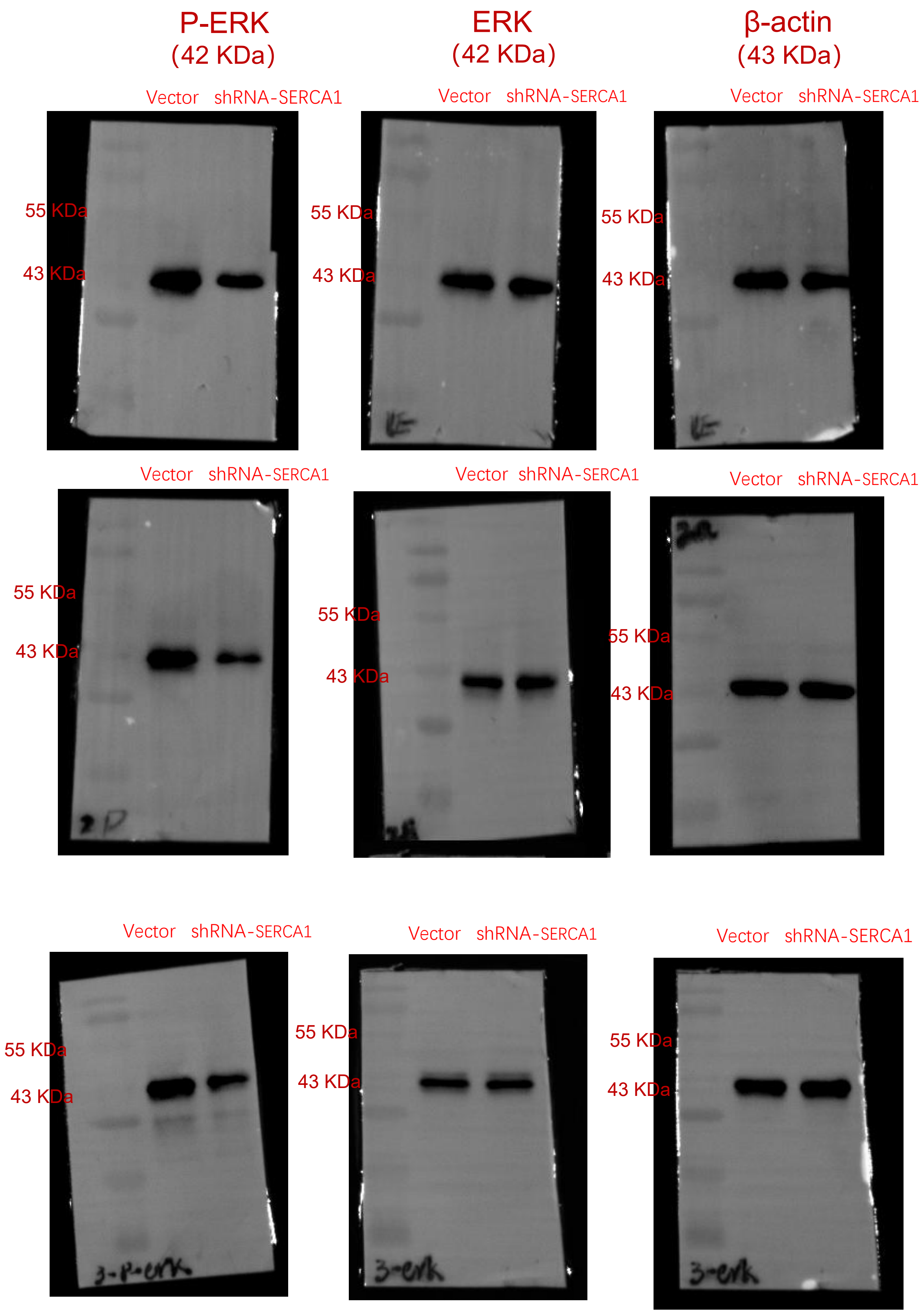

Western blot analysis total ERK and phosphorylated ERK with shRNA-treated at 3 dpa.

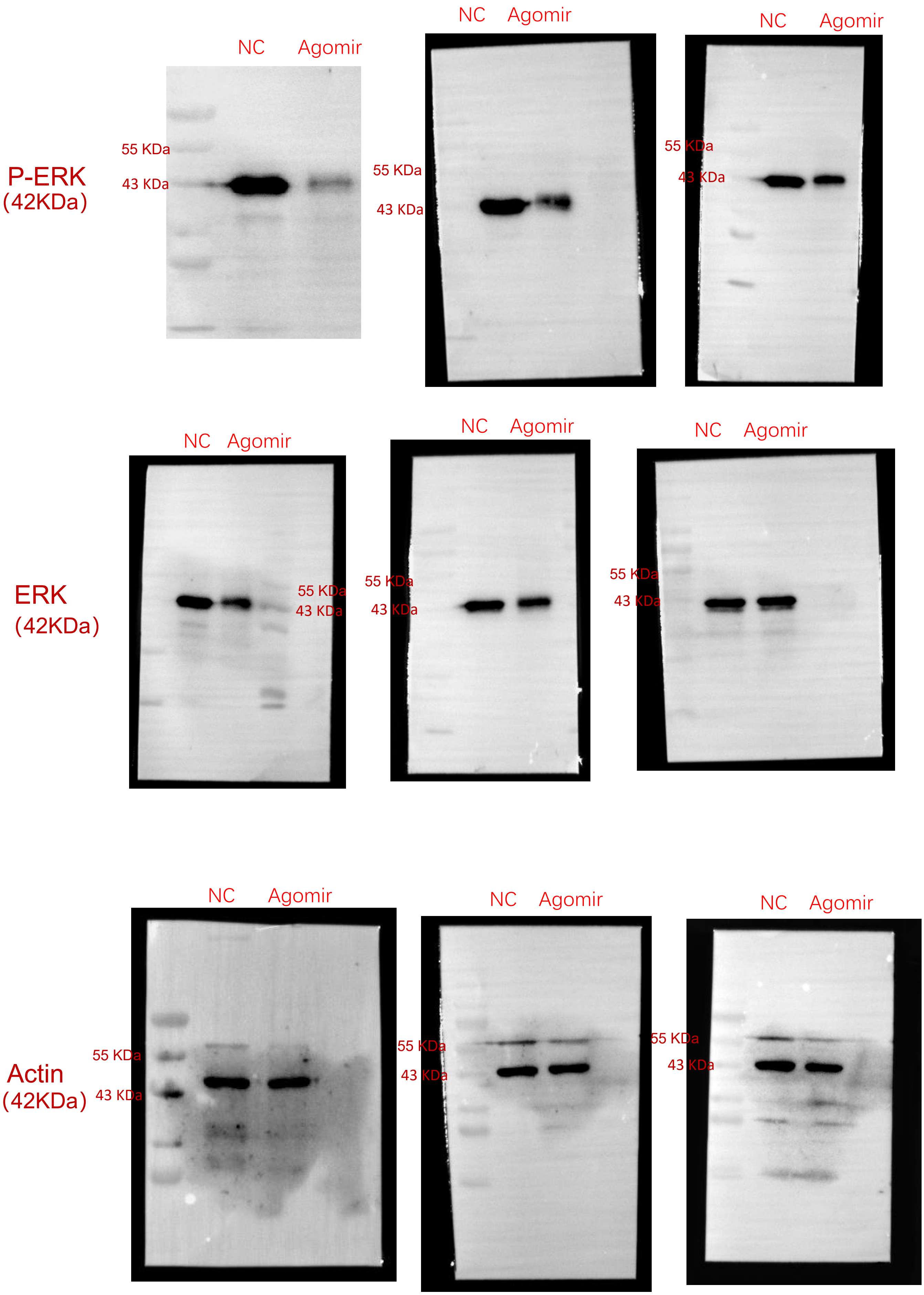

Western blot analysis total ERK and phosphorylated ERK with Agomir-treated at 3 dpa.
