# Supplementary material for: miR-20487-5p/SERCA1/MAPK/ERK Pathway Regulates Newt Limb Regeneration
Source: Biology (Basel). 2026 Jul 9;15(14):1107. doi: 10.3390/biology15141107 (PMC13404080; doi:10.3390/biology15141107)
Supplement: Supplementary file 1 [file biology-15-01107-s001.zip › biology-4387775-File S2-transcriptomic sequencing-clean data/transcriptomic sequencing-clean data/D0/NEW_Clean_Data1.fq_fastqc.zip!/T1_Clean_Data1.fq_fastqc/fastqc_report.html]

T1\_Clean\_Data1.fq FastQC Report


FastQC Report

Wed 15 Jan 2014  
T1\_Clean\_Data1.fq

## Summary

- Basic Statistics
- Per base sequence quality
- Per sequence quality scores
- Per base sequence content
- Per base GC content
- Per sequence GC content
- Per base N content
- Sequence Length Distribution
- Sequence Duplication Levels
- Overrepresented sequences
- Kmer Content

## Basic Statistics

| Measure | Value |
| --- | --- |
| Filename | T1\_Clean\_Data1.fq |
| File type | Conventional base calls |
| Encoding | Sanger / Illumina 1.9 |
| Total Sequences | 24516139 |
| Filtered Sequences | 0 |
| Sequence length | 100 |
| %GC | 47 |

## Per base sequence quality

## Per sequence quality scores

## Per base sequence content

## Per base GC content

## Per sequence GC content

## Per base N content

## Sequence Length Distribution

## Sequence Duplication Levels

## Overrepresented sequences

No overrepresented sequences

## Kmer Content

| Sequence | Count | Obs/Exp Overall | Obs/Exp Max | Max Obs/Exp Position |
| --- | --- | --- | --- | --- |
| GAAGA | 6012720 | 2.5009038 | 5.4345465 | 2 |
| CAGCA | 5341670 | 2.4541607 | 5.254024 | 1 |
| CTTCA | 5510740 | 2.254521 | 5.4369264 | 1 |
| CTGCA | 4660815 | 2.15454 | 5.120859 | 1 |
| CAGGA | 4320520 | 2.0138147 | 5.245706 | 1 |
| TCCAG | 4305570 | 1.990322 | 6.25577 | 7 |
| CTGGA | 4215600 | 1.9770131 | 5.919562 | 1 |
| CCTGG | 3640565 | 1.913271 | 5.3637114 | 1 |
| CTCCA | 4081355 | 1.8596855 | 6.2323556 | 1 |
| CTGGG | 3128720 | 1.6681377 | 5.447184 | 1 |

Produced by FastQC (version 0.10.1)
