# Supplementary material for: miR-20487-5p/SERCA1/MAPK/ERK Pathway Regulates Newt Limb Regeneration
Source: Biology (Basel). 2026 Jul 9;15(14):1107. doi: 10.3390/biology15141107 (PMC13404080; doi:10.3390/biology15141107)
Supplement: Supplementary file 1 [file biology-15-01107-s001.zip › biology-4387775-File S2-transcriptomic sequencing-clean data/transcriptomic sequencing-clean data/D0/NEW_Clean_Data2.fq_fastqc.zip!/T1_Clean_Data2.fq_fastqc/fastqc_report.html]

T1\_Clean\_Data2.fq FastQC Report


FastQC Report

Wed 15 Jan 2014  
T1\_Clean\_Data2.fq

## Summary

- Basic Statistics
- Per base sequence quality
- Per sequence quality scores
- Per base sequence content
- Per base GC content
- Per sequence GC content
- Per base N content
- Sequence Length Distribution
- Sequence Duplication Levels
- Overrepresented sequences
- Kmer Content

## Basic Statistics

| Measure | Value |
| --- | --- |
| Filename | T1\_Clean\_Data2.fq |
| File type | Conventional base calls |
| Encoding | Sanger / Illumina 1.9 |
| Total Sequences | 24516139 |
| Filtered Sequences | 0 |
| Sequence length | 100 |
| %GC | 47 |

## Per base sequence quality

## Per sequence quality scores

## Per base sequence content

## Per base GC content

## Per sequence GC content

## Per base N content

## Sequence Length Distribution

## Sequence Duplication Levels

## Overrepresented sequences

No overrepresented sequences

## Kmer Content

| Sequence | Count | Obs/Exp Overall | Obs/Exp Max | Max Obs/Exp Position |
| --- | --- | --- | --- | --- |
| GAAGA | 5958840 | 2.485024 | 5.602449 | 2 |
| CAGCA | 5296590 | 2.4425638 | 5.1842895 | 1 |
| CTTCA | 5520450 | 2.2597058 | 5.943878 | 1 |
| CTGCA | 4629390 | 2.141606 | 5.231935 | 1 |
| CAGGA | 4281885 | 1.9993857 | 5.254229 | 1 |
| TCCAG | 4264060 | 1.9726006 | 6.0620637 | 7 |
| CTGGA | 4186365 | 1.9609427 | 6.1224923 | 1 |
| CCTGG | 3649820 | 1.9142207 | 5.547361 | 1 |
| CTCCT | 4166630 | 1.9096555 | 5.327742 | 1 |
| CTCCA | 4051210 | 1.8509239 | 6.6766453 | 1 |
| CTTTG | 4413115 | 1.8348515 | 5.1680226 | 1 |
| CTGGG | 3148635 | 1.6720717 | 5.7525334 | 1 |
| CTTGG | 3427125 | 1.6103642 | 5.2197275 | 1 |

Produced by FastQC (version 0.10.1)
