# Supplementary material for: miR-20487-5p/SERCA1/MAPK/ERK Pathway Regulates Newt Limb Regeneration
Source: Biology (Basel). 2026 Jul 9;15(14):1107. doi: 10.3390/biology15141107 (PMC13404080; doi:10.3390/biology15141107)
Supplement: Supplementary file 1 [file biology-15-01107-s001.zip › biology-4387775-File S2-transcriptomic sequencing-clean data/transcriptomic sequencing-clean data/D14/D14_Clean_Data2.fq_fastqc.zip!/T4_Clean_Data2.fq_fastqc/fastqc_report.html]

T4\_Clean\_Data2.fq FastQC Report


FastQC Report

Wed 15 Jan 2014  
T4\_Clean\_Data2.fq

## Summary

- Basic Statistics
- Per base sequence quality
- Per sequence quality scores
- Per base sequence content
- Per base GC content
- Per sequence GC content
- Per base N content
- Sequence Length Distribution
- Sequence Duplication Levels
- Overrepresented sequences
- Kmer Content

## Basic Statistics

| Measure | Value |
| --- | --- |
| Filename | T4\_Clean\_Data2.fq |
| File type | Conventional base calls |
| Encoding | Sanger / Illumina 1.9 |
| Total Sequences | 27720724 |
| Filtered Sequences | 0 |
| Sequence length | 100 |
| %GC | 47 |

## Per base sequence quality

## Per sequence quality scores

## Per base sequence content

## Per base GC content

## Per sequence GC content

## Per base N content

## Sequence Length Distribution

## Sequence Duplication Levels

## Overrepresented sequences

No overrepresented sequences

## Kmer Content

| Sequence | Count | Obs/Exp Overall | Obs/Exp Max | Max Obs/Exp Position |
| --- | --- | --- | --- | --- |
| CTGGA | 4676835 | 1.9214112 | 5.2531695 | 1 |
| CTCCA | 4663730 | 1.876912 | 6.0378394 | 1 |
| CCTGG | 4037125 | 1.8366272 | 5.108635 | 1 |
| CTGGG | 3618265 | 1.6631371 | 5.2549224 | 1 |

Produced by FastQC (version 0.10.1)
