# Supplementary material for: miR-20487-5p/SERCA1/MAPK/ERK Pathway Regulates Newt Limb Regeneration
Source: Biology (Basel). 2026 Jul 9;15(14):1107. doi: 10.3390/biology15141107 (PMC13404080; doi:10.3390/biology15141107)
Supplement: Supplementary file 1 [file biology-15-01107-s001.zip › biology-4387775-File S2-transcriptomic sequencing-clean data/transcriptomic sequencing-clean data/D42/D42_Clean_Data2.fq_fastqc.zip!/T6_Clean_Data2.fq_fastqc/fastqc_report.html]

T6\_Clean\_Data2.fq FastQC Report


FastQC Report

Wed 15 Jan 2014  
T6\_Clean\_Data2.fq

## Summary

- Basic Statistics
- Per base sequence quality
- Per sequence quality scores
- Per base sequence content
- Per base GC content
- Per sequence GC content
- Per base N content
- Sequence Length Distribution
- Sequence Duplication Levels
- Overrepresented sequences
- Kmer Content

## Basic Statistics

| Measure | Value |
| --- | --- |
| Filename | T6\_Clean\_Data2.fq |
| File type | Conventional base calls |
| Encoding | Sanger / Illumina 1.9 |
| Total Sequences | 25566015 |
| Filtered Sequences | 0 |
| Sequence length | 100 |
| %GC | 46 |

## Per base sequence quality

## Per sequence quality scores

## Per base sequence content

## Per base GC content

## Per sequence GC content

## Per base N content

## Sequence Length Distribution

## Sequence Duplication Levels

## Overrepresented sequences

No overrepresented sequences

## Kmer Content

| Sequence | Count | Obs/Exp Overall | Obs/Exp Max | Max Obs/Exp Position |
| --- | --- | --- | --- | --- |
| CTTCA | 5581035 | 2.194054 | 5.227902 | 1 |
| CTGCA | 4918510 | 2.1883047 | 5.0104084 | 1 |
| CTCCT | 4231990 | 1.8685676 | 5.2726874 | 1 |
| CTGGA | 4153370 | 1.865476 | 5.318331 | 1 |
| CTCCA | 4165850 | 1.8359591 | 6.2866135 | 1 |
| CCTGG | 3603825 | 1.8179603 | 5.112621 | 1 |
| CTGGG | 3291735 | 1.6763332 | 5.603988 | 1 |

Produced by FastQC (version 0.10.1)
