# Supplementary material for: miR-20487-5p/SERCA1/MAPK/ERK Pathway Regulates Newt Limb Regeneration
Source: Biology (Basel). 2026 Jul 9;15(14):1107. doi: 10.3390/biology15141107 (PMC13404080; doi:10.3390/biology15141107)
Supplement: Supplementary file 1 [file biology-15-01107-s001.zip › biology-4387775-File S2-transcriptomic sequencing-clean data/transcriptomic sequencing-clean data/D7/D7_Clean_Data2.fq_fastqc.zip!/T3_Clean_Data2.fq_fastqc/fastqc_report.html]

T3\_Clean\_Data2.fq FastQC Report


FastQC Report

Wed 15 Jan 2014  
T3\_Clean\_Data2.fq

## Summary

- Basic Statistics
- Per base sequence quality
- Per sequence quality scores
- Per base sequence content
- Per base GC content
- Per sequence GC content
- Per base N content
- Sequence Length Distribution
- Sequence Duplication Levels
- Overrepresented sequences
- Kmer Content

## Basic Statistics

| Measure | Value |
| --- | --- |
| Filename | T3\_Clean\_Data2.fq |
| File type | Conventional base calls |
| Encoding | Sanger / Illumina 1.9 |
| Total Sequences | 28670611 |
| Filtered Sequences | 0 |
| Sequence length | 100 |
| %GC | 47 |

## Per base sequence quality

## Per sequence quality scores

## Per base sequence content

## Per base GC content

## Per sequence GC content

## Per base N content

## Sequence Length Distribution

## Sequence Duplication Levels

## Overrepresented sequences

No overrepresented sequences

## Kmer Content

| Sequence | Count | Obs/Exp Overall | Obs/Exp Max | Max Obs/Exp Position |
| --- | --- | --- | --- | --- |
| CAGCA | 6466265 | 2.5428543 | 5.142876 | 1 |
| CTGCA | 5685245 | 2.244879 | 5.010422 | 1 |
| CTTCA | 6234410 | 2.192251 | 5.091713 | 1 |
| TCCAG | 4832015 | 1.9079721 | 5.079676 | 7 |
| CTGGA | 4743360 | 1.8917657 | 5.5488863 | 1 |
| CCTGG | 4156440 | 1.8505032 | 5.1368856 | 1 |
| CTCCA | 4716130 | 1.8437073 | 6.182632 | 1 |
| CTGGG | 3827115 | 1.720986 | 5.661164 | 1 |

Produced by FastQC (version 0.10.1)
